# Interictal epileptiform discharges show distinct spatiotemporal and morphological patterns across wake and sleep

**DOI:** 10.1093/braincomms/fcac183

**Published:** 2022-07-18

**Authors:** Amal Fouad, Hamed Azizollahi, Jean-Eudes Le Douget, François-Xavier Lejeune, Mario Valderrama, Liliana Mayor, Vincent Navarro, Michel Le Van Quyen

**Affiliations:** Bioelectrics Lab, Paris Brain Institute (ICM Institut du Cerveau), (UMRS 1127, CNRS UMR 7225), Pitié-Salpêtriere Hospital, 75013 Paris, France; Department of Neurology, Faculty of medicine, Ain-Shams University, Cairo, Egypt; Bioelectrics Lab, Paris Brain Institute (ICM Institut du Cerveau), (UMRS 1127, CNRS UMR 7225), Pitié-Salpêtriere Hospital, 75013 Paris, France; Bioserenity, Paris Brain Institute (ICM Institut du Cerveau), 75013 Paris, France; Bioelectrics Lab, Paris Brain Institute (ICM Institut du Cerveau), (UMRS 1127, CNRS UMR 7225), Pitié-Salpêtriere Hospital, 75013 Paris, France; Bioserenity, Paris Brain Institute (ICM Institut du Cerveau), 75013 Paris, France; Sorbonne University, Paris, France; Paris Brain Institute (ICM Institut du Cerveau), AP-HP, INSERM, CNRS, University Hospital Pitié-Salpêtrière, 75013 Paris, France; Paris Brain Institute's Data and Analysis Core (ICM Institut du Cerveau), University Hospital Pitié-Salpêtrière, 75013 Paris, France; Department of Biomedical Engineering, University of los Andes, Bogotá, Colombia; Roosevelt Institute, Bogotá, Colombia; Sorbonne University, Paris, France; Paris Brain Institute (ICM Institut du Cerveau), AP-HP, INSERM, CNRS, University Hospital Pitié-Salpêtrière, 75013 Paris, France; Epileptology Unit, AP-HP Pitié-Salpêtrière Hospital, 75013 Paris, France; Bioelectrics Lab, Paris Brain Institute (ICM Institut du Cerveau), (UMRS 1127, CNRS UMR 7225), Pitié-Salpêtriere Hospital, 75013 Paris, France; Sorbonne University, Paris, France; Laboratoire D’Imagerie Biomédicale, (INSERM U1146, UMR7371, CNRS), Sorbonne University, Paris, France

**Keywords:** interictal epileptiform discharges, sleep, seizure onset

## Abstract

Presurgical evaluation of mesial temporal and neocortical focal pharmacoresistant epilepsy patients using intracranial EEG recordings has led to the generation of extensive data on interictal epileptiform discharges, located within or remotely from seizure onset zones. In this study, we used this data to investigate how interictal epileptiform discharges are modulated and how their spatial distribution changes during wake and sleep and analysed the relationship between these discharge events and seizure onset zones. Preoperative evaluation data from 11 adult patients with focal pharmacoresistant epilepsy were extracted from the Epilepsiae database. Interictal epileptiform discharges were automatically detected during wakefulness and over several hours of continuous seizure-free sleep (total duration of EEG recordings:106.7 h; mean per patient: 9.7 h), and analysed across four brain areas (mesial temporal, lateral neocortical, basal cortical and the temporal pole). Sleep stages were classified manually from scalp EEG. Discharge events were characterized according to their rate and morphology (amplitude, sharpness and duration). Eight patients had a seizure onset zone over mesial areas and three patients over lateral neocortical areas. Overall, discharge rates varied across brain areas during wakefulness and sleep [*wake/sleep stages* × *brain areas interaction*; Wald *χ*^2^(df = 6) = 31.1, *P* < 0.0001]. N2–N3 non-rapid eye movement sleep increased interictal epileptiform discharges in mesial areas compared with wakefulness and rapid eye movement sleep (*P* < 0.0001), and to other areas (*P* < 0.0001 for all comparisons). This mesial pattern was observed both within and outside of seizure onset zones. During wakefulness, the rate of interictal epileptiform discharges was significantly higher than during N2–N3 non-rapid eye movement sleep (*P* = 0.04), and rapid eye movement sleep (*P* = 0.01) in lateral neocortical areas (referred to as lateral neocortical pattern), a finding that was more pronounced in seizures onset zones (*P* = 0.004). The morphological characteristics of the discharge events were modulated during wakefulness and sleep stages across brain areas. The effect of seizure onset zones on discharge morphology was conditioned by brain area and was particularly marked in temporal pole areas. Our analysis of discharge patterns in relation to cerebral localization, vigilance state and the anatomical affiliation of seizure onset zones revealed the global and local aspects of the complex relationship between interictal discharges, sleep and seizure onset zones. This novel approach may lead to a better understanding of cognitive decline and responses to therapy, as well as to adaptation of surgical interventions for epileptic patients.

## Teaser

Fouad et al. report that seizure-free wakefulness and sleep stages, as well as seizure onset zones, modulate interictal discharge rates and morphology in focal epilepsy. The mesial discharges were facilitated by deep sleep, while the neocortical discharges were enhanced by wakefulness. Seizure onset zones influenced discharge rates and morphology.

## Graphical Abstract

**Graphical Abstract:**
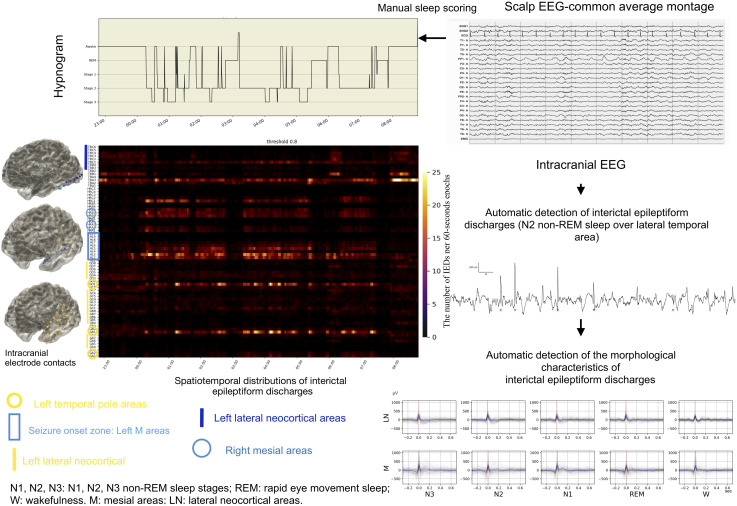

## Introduction

Interictal epileptiform discharges (IEDs) are paroxysmal events that are known to be associated with seizures and epilepsy.^1,2^ Morphologically, IEDs, referred to as spikes, spike-wave complexes, sharp waves and sharp-wave complexes, are considered as pathological changes on the EEG. Despite their common use to describe IEDs, little is known about how these events are modulated across multiple brain areas during sleep.

A promising approach for investigating IEDs during sleep has emerged from the analysis of the long-term intracranial EEGs (iEEGs) performed as a part of the presurgical evaluation of patients with focal pharmacoresistant epilepsy.^3^ These long-term iEEGs register ictal events and IEDs through multiple spontaneous wake and sleep periods, and thus generate a wealth of data that can be used to track IEDs on multiple scales. Recently, automated techniques have made it possible to extract and analyse IED features.

Analyses of long-term iEEG data have already been used to show that the spatial distribution of IEDs varies over time, with clear fluctuations being observed in an association with sleep.^4,5^ Previous studies using surface EEG and iEEG have also highlighted the quantitative enhancement of IEDs during sleep in focal temporal epilepsy, particularly during non-rapid eye movement (non-REM) sleep.^4,6^ Furthermore, analysis of iEEG from patients with focal epilepsy has led to the reconceptualization of sleep as a locally modulated state,^7–9^ and the coexistence of local sleep-like and wake-like patterns in different brain areas found to characterize certain sleep disorders.^10^

IEDs have been found to extend beyond the epileptic focus and to be associated with brain network changes in humans,^11–13^ and in animal models of focal epilepsy.^14^ Recent research has shown that IED events and seizures show co-occurring rhythmicity,^15,16^ suggesting that they are not independent processes.

In this study, we investigated whether analysis of automatically detected IEDs, and their morphological attributes, during wakefulness and sleep could provide valuable insights into the dynamics of interictal activity across brain areas and in relation to seizure onset zones (SOZs).

## Materials and methods

This study was conducted using data from the Epilepsiae database. This European database contains records of long-term iEEGs and/or scalp EEGs registered during the preoperative evaluation of 275 patients with focal pharmacoresistant epilepsy.^17^

### Patient selection

The study evaluated consecutive EEG recordings from adult patients who had undergone combined and concomitant iEEG and scalp EEG. Only data from patients who had at least one continuous night free of clinical seizures, registered at least 24 h after electrode implantation, and had at least one iEEG-confirmed clinical seizure with a focal onset were included.

Approval for the study was obtained from the bioethics commissions relevant for Klatt *et al*.^17^

### Intracranial and scalp EEG recordings

Recordings were acquired using a Neurofile NT digital video EEG system with 128 channels and a sampling rate of 1024 Hz. Scalp EEGs were obtained using the 10–20 system, including two electrooculogram channels, and at least 19 EEG channels. iEEGs were recorded by subdural cortical grids, subdural strips and multiple-contact depth macroelectrodes. The depth macroelectrodes were primarily employed to sample the hippocampi.

Before analysis, the EEG signal was high-pass filtered at 0.5 Hz, low-pass filtered at 70 Hz and a notch filter was applied to remove 50 Hz noise (49–51 Hz).

### Electrode localization and brain-area assignment

The Epilepsiae database contains the three-dimensional coordinates of the intracranial electrodes based on the Montreal Neurologic Institute coordinate system,^17^ as well as functions to calculate the equally widespread Talairach coordinates. These coordinates were used to determine the anatomical location of each electrode contact according to Brodmann localization,^18^ and subsequent coregistration to a Matlab-created virtual brain template. After that, electrodes were then assigned to one of four defined brain areas: (i) mesial temporal areas (M; hippocampus, amygdala, uncus, entorhinal gyrus and parahippocampal gyrus), (ii) the temporal pole (TP), (iii) basal cortical areas (BC; basal part of inferior temporal gyrus, fusiform gyrus) and (iv) lateral neocortical areas (LN; lateral frontal, lateral parietal, lateral occipital and lateral temporal areas).

### Identification of SOZs and patient groups

Clinical seizures were identified using iEEG recordings. The Epilepsiae database contains the electrographic timestamps and the names of the intracranial electrode contacts associated with SOZ, based on the earliest electrographic change. This information was then used to extract SOZ electrode contacts for each clinical seizure. Patients were subsequently divided into two groups: a mesial group (M-SOZ group), which had SOZ-associated electrode contacts in M areas, and a LN group (LN-SOZ group), which had SOZ-associated electrode contacts in LN areas.

Based on previous descriptions of the anatomical network of the TP, its connection with mesial structures, and its connections with the neocortex,^19,20^ patients who had SOZs detected over TP areas and were associated with either SOZ-associated M areas or SOZ-associated LN areas were classified into M-SOZ or LN-SOZ groups, respectively.

### Selection of seizure-free nights and sleep staging

For each patient, the number and order of recorded nights were verified. First-night EEG data were not analysed to avoid sleep disturbances caused by the so-called first-night effect.^21^

The following criteria were used to identify seizure-free nights and associated periods of wakefulness (wake epochs recorded immediately before and after the period of seizure-free sleep): (i) continuous sequential multi-hour wake/sleep epochs and (ii) a 6-h delay between the seizure-free sleep and the occurrence of clinical seizures.

We scored sleep using scalp EEG recordings corresponding to selected seizure-free nights and wakefulness. According to the American Academy of Sleep Medicine,^22^ sleep was manually scored in 30-s epochs and classified as non-REM sleep stages (N1, N2, or N3) or REM sleep. We examined scalp EEGs to determine whether there were any major anomalies in the background activity that could have interfered with sleep staging.

### Detection of IEDs from iEEG recordings

Following sleep staging, the iEEG corresponding to selected wake/sleep epochs was visually examined and then assessed for IEDs using a validated automatic detection method.

For each patient, the manual screening procedure was to visualize at least 2 min of iEEG data (on 30-s epochs) from the wake and each sleep stage of the first wake/sleep cycle using a common averaged montage. True IEDs were identified using the revised glossary of terms for clinical EEGs.^23^ Visual screening also allowed the exclusion of intracranial electrode contacts with potential physiological rhythms resembling IEDs, or artefacts, as well as those with no IED activity. A spike’s positive polarity was not an exclusion criterion.

The validated automatic IED detection method used in this study, as well as the procedure for determining patient-specific thresholds, have been previously described.^24^ In brief, data were divided into 1-hour sections before being processed using common average montaging, bandpass filtering (0.5–70 Hz) and notch-filtering (49–51 Hz). The following steps were then performed: (i) peaks in each signal were identified using a dual amplitude and slope threshold; (ii) data were extracted from a 1-s window around each peak and (iii) features were extracted from the data using discrete wavelet decomposition and entered into a random forest classifier, trained and cross-validated on a set of iEEG segments with 3000 manually annotated IEDs. The classifier then returned a probabilistic output for each peak identified in step (1), providing a confidence estimate for the IED detections.

For all patients, the classifier output threshold was set at 0.8; this threshold was chosen to promote a high level of precision for IED detection (i.e. a high ratio of correct IED detections among all detections). To assess the performance and the precision, a random selection of 50 automatically detected IEDs for each patient was visually validated by two neurologists (10 from N1, N2 and N3 sleep stages, and 10 from the wake period). True-positive and false-positive rates were then calculated.

### Identification of the morphological features of the IEDs

IED candidates identified by the automatic detection method were then analysed using an automatic method developed earlier by Liu *et al*.^25^ to extract the following morphological features: (i) spike or sharp-wave amplitude, (ii) spike or sharp-wave sharpness, (iii) spike or sharp-wave duration, (iv) after-spike or after-sharp-wave-slow-wave amplitude and (v) after-spike or after-sharp-wave-slow-wave duration. In the description of our analysis, we refer to spikes and sharp waves as ‘spikes’, and after-spike or after-sharp-wave-slow-waves as ‘slow waves’.

### Change in the spatial distribution of IEDs through wake and sleep

To examine whether the spatial distribution of IEDs was modulated during wakefulness and sleep, linear mixed models (LMMs) were used to compare IED rates (mean number of IEDs per min) throughout wakefulness and sleep across brain areas.

IED rates were calculated using the number of automatically detected IEDs per 30-s iEEG epoch, averaged for each brain area (region of interest), and then averaged through each wake and sleep stage, and for each patient in all possible combinations. Based on the fitted LMMs, the significance of the main and interaction effects was determined using Type II Wald *χ*^2^ tests. The null hypothesis indicated that IED rates were comparable through wake and sleep across brain areas. As a result, significant differences implied that spatial variability of the rate of IEDs was wake- or sleep-dependent. When a significant effect was found, Tukey’s *post hoc* pairwise comparisons were performed. For simplicity, N1 sleep stage was excluded from Tukey’s *post hoc* analysis; then N2 and N3 non-REM sleep stages were combined (N2–N3 non-REM sleep). The details of the tests are available in the ‘Statistical analysis’ section.

### Change in the IED rates across SOZs and non-SOZs

To study the influence of SOZs on IEDs, the rate of IEDs was compared in SOZs and non-SOZs-involved brain areas during the wake and sleep using LMMs with Type II Wald *χ*^2^ tests. Tukey’s *post hoc* pairwise comparisons were used to analyse significant effects.

### Spatiotemporal distribution of IEDs across patient groups

To assess whether variability in IEDs differed between patient groups, SOZs-involved brain areas (M ± TP in M-SOZ group, and LN ± TP in LN-SOZ group), and non-SOZs were analysed for each patient group using LMMs with Type II Wald *χ*^2^ tests. Significant effects were further analysed using Tukey’s *post hoc* pairwise comparisons.

### Changes in the morphological characteristics of IEDs

To describe the variability of the morphological characteristics of IEDs, averaged data for each characteristic were analysed across brain areas during wake and sleep as well as in SOZ and non-SOZ-involved areas.

#### Morphological characteristics of IEDs across brain areas during wake and sleep

Differences in the morphological parameters (spike duration, spike sharpness, spike amplitude, slow-wave amplitude, slow-wave duration) were assessed by LMMs. In each model, the following factors: anatomical location (area) (LN, TP, M), SOZ (SOZ, non-SOZ), wake and sleep stages (N1, N2–N3, W, REM) and their interaction terms were regarded as fixed effects. Furthermore, the type of electrodes (strip, grid, depth) was added in the model for covariate adjustment to test for possible effect. Significance for the main effects and all two- and three-way interactions of location (area), SOZ, sleep stages were analysed based on Type II Wald *χ*^2^ tests; *post hoc* pairwise comparisons were carried out on a significant effect of the main factors or higher-level interactions with Tukey’s method. As there were no SOZ in BC areas, analysis involving SOZ/non-SOZ was conducted after exclusion of BC. Analysis was performed for all sleep stages separately and for N2–N3 combined. The level of statistical significance was set at *P* < 0.05 for all tests.

#### SOZs and the morphological characteristics of IEDs

For each morphological characteristic, a LMM was fitted to test whether there was a significant main effect of the anatomical location (area), SOZ and wake and sleep stage, as well as for the presence of significant interaction effects between these conditions.

### Statistical analysis and software

All statistical analyses were conducted in R version 3.5.2.^26^ To test for parameters of interest, all LMMs were fitted using restricted maximum-likelihood estimation from the lmer function in the *lme4* package (v1.1-21).^27^

In all models, the factors conditions (areas, SOZ, wake/sleep), and their interaction terms were regarded as fixed effects. The patient identifier was assigned as a random effect (intercept) to account for repeated measures by the subject.

Based on LMMs, significant results were determined by Type II Wald *χ*^2^ tests calculated with ANOVA function in the *car* package (v3.0-7). *Post hoc* pairwise comparisons were carried out when either a significant main factor effect or interaction effect was found, with the *emmeans* package (v1.4.5). *P*-values resulting from the *post hoc* pairwise comparisons were obtained using Kenward-Roger’s approximation for degrees of freedom (df), and after adjustment with the Tukey’s method to account for multiple testing. Furthermore, the directions of the difference were indicated based on the sign of the estimated marginal mean differences from *emmeans* (i.e. difference of the group means estimated by the LMMs).

In the statistical description, Wald *χ*^2^ Type II tests are referred to as Wald *χ*^2^ tests with the values of test-statistics, df and *P*-values.

For the sake of readability, *post hoc* pairwise comparisons tests will be referred to as Tukey’s *post hoc* analysis, with only adjusted *P*-values.

Before analysing the morphological characteristics of IEDs, a limited number of abnormally large values exceeding five times the interquartile range over the median overall channels were removed to reduce the influence of extreme outliers. The rates and morphological characteristics of the IEDs are presented as mean ± standard error of the mean. The level of statistical significance was set at *P* < 0.05 for all tests.

To account for electrode type effects on morphological parameters, further analysis with electrode type adjustment was performed. The findings were then compared with those without electrode type adjustment.

Automatic detection and processing of IEDs were performed using Python 3.7, with NumPy, Scipy, Scikit-learn, and Pandas libraries. Signal visualizations and IED distribution heat maps were made using the Matplotlib package: higher frequencies were displayed in brighter colours (red/orange) and low frequencies in darker colours (blue/green). Electrode contacts were displayed on a MATLAB-created virtual brain template using MATLAB R2016.

### Data availability

Data can be made available upon reasonable request. EEG datasets are available in the Epilepsiae repository, http://www.epilepsiae.eu/.

## Results

### Clinical characteristics and IED detection

Twelve patients (five males and eight females, aged 17–63 years, mean and SD 33.63 ± 14.69 years) fulfilled the inclusion criteria. After a visual review of SOZ, one patient was excluded due to the absence of the earliest electrographic changes.

SOZs were found in the Mesial areas of eight patients (M-SOZ group) and in the LN areas of three patients (LN-SOZ group). The clinical and demographic characteristics of the remaining 11 patients are summarized in Table 1.

**Table 1:** Clinical, demographic and iEEG characteristics of the study population

Patient number	Age at onset/recording	Gender	SOZ area	Pathology	Number of 30-s epochs/number of automatically detected IEDs					Number of electrode contacts per brain area			
					N3	N2	N1	REM	Wake	Lateral neocortical	Basal cortical	Temporal pole	Mesial temporal
**1**	5/21	Male	Right M	No visible lesion	130/2232	424/6941	137/2403	51/244	590/8089	10	9	6	11
**2**	1/17	Male	Left M	Left CD and left HS	213/14 075	456/20 461	32/1506	114/5356	376/18 942	27	17	12	12
**3**	11/18	Male	Bilateral M (+ both TPs)	Temporal CD	295/17 249	743/23 470	93/2072	49/583	306/3989	43	4	11	30
**4**	30/63	Female	Bilateral M (+ left TP)	Tumour at the base of the skull	131/3439	433/8745	26/346	138/690	474/3215	6	11	10	14
**5**	18/23	Male	Left M (+ left TP)	Left temporal CD	54/1228	102/2515	101/947	30/34	368/1101	18	5	8	10
**6**	10/47	Female	Right M (+ right TP)	Right temporal CD, right HS	251/4748	501/10 232	56/941	134/1945	278/3357	7	3	3	10
**7**	22/32	Female	Right M	Right hippocampus Malrotation and right CD	338/34 401	737/37 414	38/1285	114/3084	218/8239	26	5	11	8
**8**	1/32	Female	Left M	Left CD and HS	101/2906	535/23 165	22/786	147/1861	396/10 187	28	4	6	16
**9**	16/42	Male	Right LN (+ right TP)	Right CD	297/9005	435/14 039	37/960	173/659	364/8627	8	14	4	12
**10**	7/48	Female	Left LN	Not available	208/5476	161/7871	43/4979	0/0	254/34 385	76	0	0	0
**11**	10/27	Female	Left LN (+ left TP)	Left CD	233/3541	444/8438	49/1059	111/1945	261/9468	31	9	5	0

BC, basal cortical areas; CD, cortical dysplasia; HS, hippocampal sclerosis; IED, interictal epileptiform discharges; LN, lateral neocortical areas; M, mesial temporal areas; N1, N2 and N3, non-REM sleep stages; REM, rapid eye movement sleep; SOZ, seizure onset zone; TP, temporal pole.

Twenty-seven electrode contacts were excluded: 8 in BC and 19 in LN, due to the presence of physiological rhythms that resembled IEDs or artefacts, or because they did not record IED activity.

All subsequent analyses were performed on a total of 560 intracranial electrode contacts.

Table 1 shows the number of intracranial electrode contacts used for each patient, according to brain area covered. The distribution of electrode types varied between patients (Supplementary Fig. 1). The absolute number of electrode contacts per electrode type, as well as their percentages per area and patient, are shown in Supplementary Fig. 2.

The number of SOZ-associated electrode contacts differed between patients (Table 2).

**Table 2:** Number of electrode contacts in SOZ/non-SOZs

	Total number of electrode contacts (Total non-SOZ/Total SOZ)				
Group/Subject ID	Basal neocortical	Lateral neocortical	Mesial	Temporal pole	Total
M-1	9 (9/0)	10 (10/0)	11 (9/2)	6 (3/3)	36 (31/5)
M-2	17 (17/0)	27 (27/0)	12 (5/7)	12 (12/0)	68 (61/7)
M-3	4 (4/0)	43 (43/0)	30 (22/8)	11 (5/6)	88 (74/14)
M-4	11 (11/0)	6 (6/0)	14 (0/14)	10 (2/8)	41 (19/22)
M-5	5 (5/0)	18 (18/0)	10 (10/0)	8 (0/8)	41 (33/8)
M-6	3 (3/0)	7 (7/0)	10 (6/4)	3 (3/0)	23 (19/4)
M-7	5 (5/0)	26 (26/0)	8 (7/1)	11 (11/0)	50 (49/1)
M-8	4 (4/0)	28 (28/0)	16 (8/8)	6 (6/0)	54 (46/8)
LN-9	14 (14/0)	8 (6/2)	12(12/0)	4 (1/3)	38 (33/5)
LN-10	0 (0/0)	76 (61/25)	0 (0/0)	0 (0/0)	76 (61/15)
LN-11	9 (9/0)	31 (27/4)	0 (0/0)	5 (3/2)	45 (39/6)

For each subject, the table shows the distribution of electrode contacts across the four brain areas. The numbers in parentheses indicate, respectively, the number of non-SOZ electrode contacts and the number of SOZ electrode contacts. SOZ, seizure onset zone; non-SOZ, non-seizure onset zone; M, M-SOZ group; LN, LN-SOZ group.

A total of 95 electrode contacts recorded SOZs, while 465 recorded non-SOZ areas. Four hundred and one contacts were analysed from M-SOZ group, of which 332 covered non-SOZs and 69 covered SOZs. In the LN-SOZ group, there were 133 non-SOZ contacts and 26 SOZ contacts (total electrode contacts: 159).

The total duration of all EEG recordings was 106.7 h (mean per patient: 9.7 ± 2.3 h). Wake and sleep epochs were identified in all patients, except for Patient 10, for whom sufficient epochs of REM sleep were not observed. Table 1 shows the number of 30-s iEEG epochs for each wake and sleep stage.

**Figure 1:**
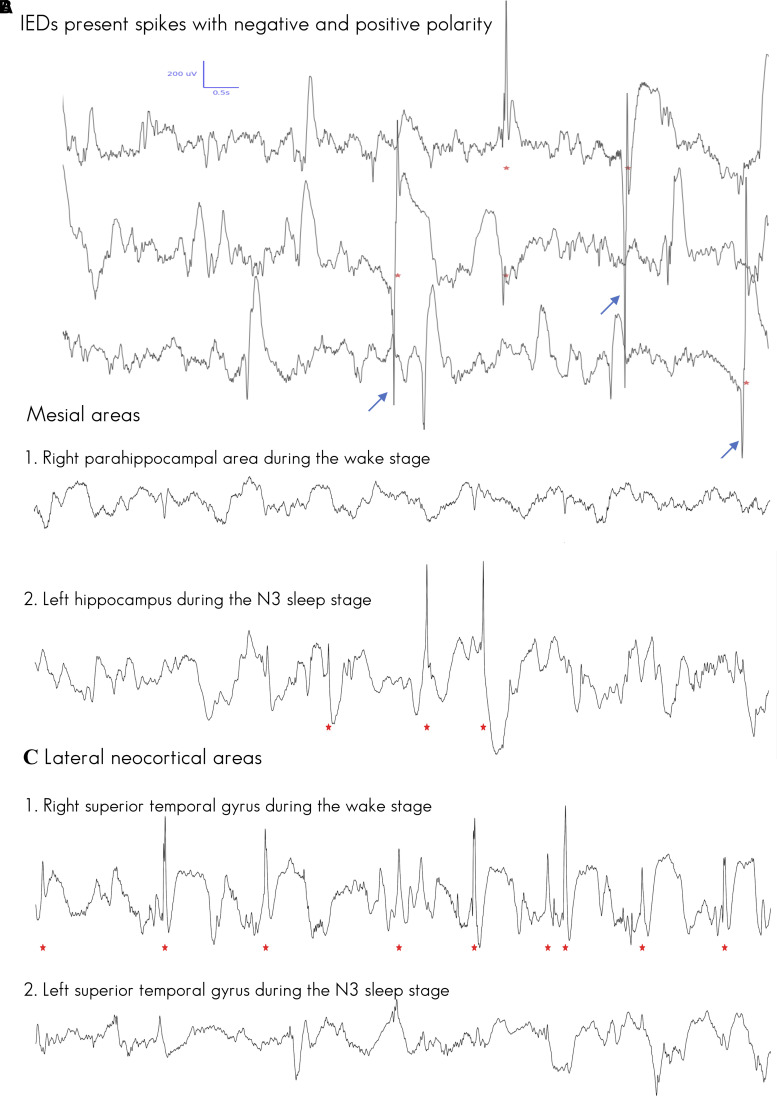
**Automatic detection of IEDs using iEEG recordings**. IEDs identified using the automatic detection method are marked by a red star. The detection threshold was set at 0.8 for all analyses. (**A)** A sample of automatically detected spikes with positive polarity (pointing downward, marked by blue flash), and negative polarity (pointing upwards) detected in right hippocampus. (**B**) Two mesial areas with varying IED activity during wake and N–N3 non-sleep. The right parahippocampal area (Patient 9) showed no detected IED during wakefulness. In contrast, during the N2–N3 non-sleep period, the left hippocampus (Patient 8) demonstrated detectable IED activity. (**C**) Two LN areas with varying IED activity during wake and N2–N3 non-sleep. The right superior temporal gyrus (Patient 9) showed frequent IEDs during wakefulness. In contrast, during the N2–N3 non-sleep period, the left superior temporal gyrus (Patient 8) demonstrated no obvious IED activity.

Globally, the automatic detection method had a precision of 92%. Precision was stable across the wake and sleep stages (range: 90–93%). Supplementary Table 1 shows the levels of precision obtained for each patient and sleep stage, and Fig. 1 illustrates an example of the performance of the automatic detection method. Table 1 indicates the number of detected IEDs during the wake and sleep stages for each patient.

Overall, 12 802 30-s epochs were analysed (mean per patient:164 ± 274) and a total of 404 875 IEDs were detected (mean number per patient: 36 807 ± 22 931). In Supplementary Table 2, the number of IEDs is shown by brain areas in both patient groups.

### Sleep facilitates the occurrence of IEDs, and REM sleep is associated with lower rates of IEDs

**Figure 2:**
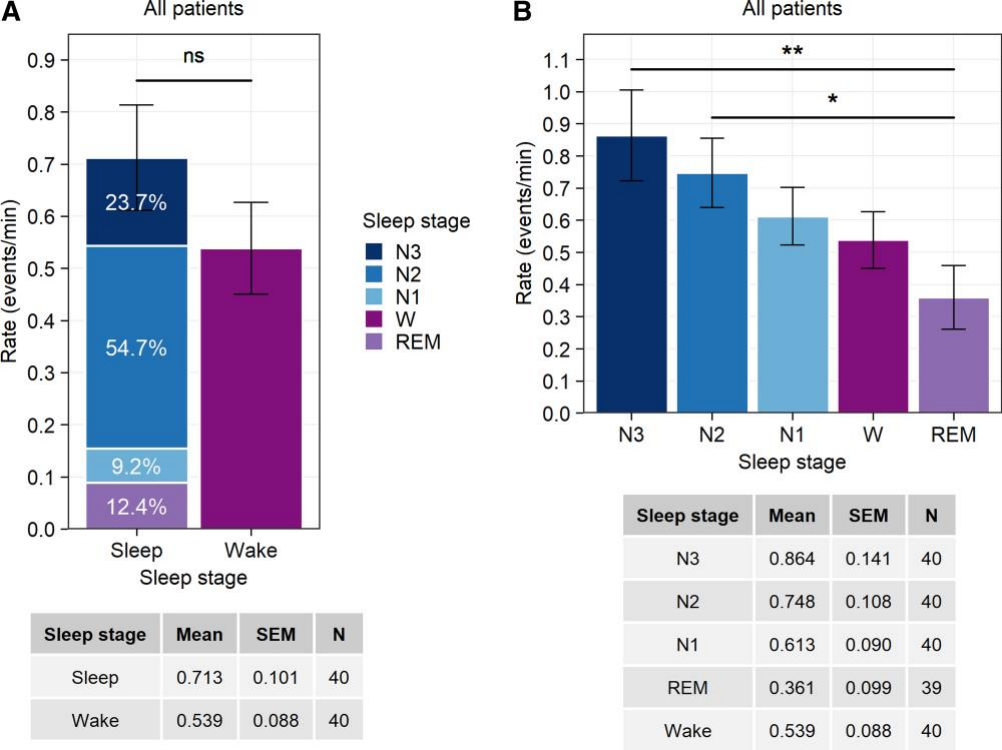
**The rate of IEDs during the wake and sleep stages (N1, N2, N3 non-REM and REM)**. The descriptive statistics (mean, standard error of the mean and number of values) are shown in the tables below the figure. (**A**) The rate of IEDs during wake and sleep. For sleep, the colour bar was subdivided to represent the percentage of the weighted duration of average rate of IEDs for each sleep stage (N3, N2, N1 and REM) in relation to the total duration of sleep. The difference between wake and sleep is non-significant [Wald *χ*^2^(1), *P* = 0.14]. *N* are the number of the values extracted from the average rate of IEDs across brain areas and subjects. Each value is a product of the average values per brain area and per subject. The number of values (*N*) for wake and sleep: BC, *n* = 10 areas; LN, *n* = 11 areas; M, *n* = 9 areas; TP, *n* = 10 areas (refer to Table 2 for more details). (**B**) The variability of the rate of IEDs during wake and different sleep stages. The number of values (*N*) per wake and per sleep stage: BC, *n* = 10 areas; LN, *n* = 11 areas; M, *n* = 9 areas; TP, *n* = 10 areas. The rate of IEDs varied throughout wake and different sleep stages [Wald *χ*^2^(4), *P* = 0.003]. When compared with N2 and N3, REM sleep had a significant lower rate of IEDs (Tukey’s *post hoc*; *REM sleep versus N2-non-REM sleep: *P* = 0.046; **REM sleep versus N3 non-REM sleep: *P* = 0.003). BC, basal cortical; TP, temporal pole; M, mesial; LN, lateral neocortical. Significance of the differences is indicated by asterisks: **P < 0.01, *P < 0.05, ns, non-significant. The values for wake and each sleep stage are presented as mean ± standard error of the mean (SEM).

Figure 2A demonstrates the rate of IEDs during wake and sleep, as well as their descriptive statistics. The difference between wake and sleep was not statistically significant (Wald *χ*^2^(df = 1), *P* = 0.14). Figure 2B shows the variability of the rate of IEDs throughout the wake and sleep stages. We found that REM sleep had a significantly lower rate of IEDs when compared with N2 and N3 non-REM sleep.

### Wake and sleep modulate the spatial distribution of IEDs across brain areas

**Figure 3:**
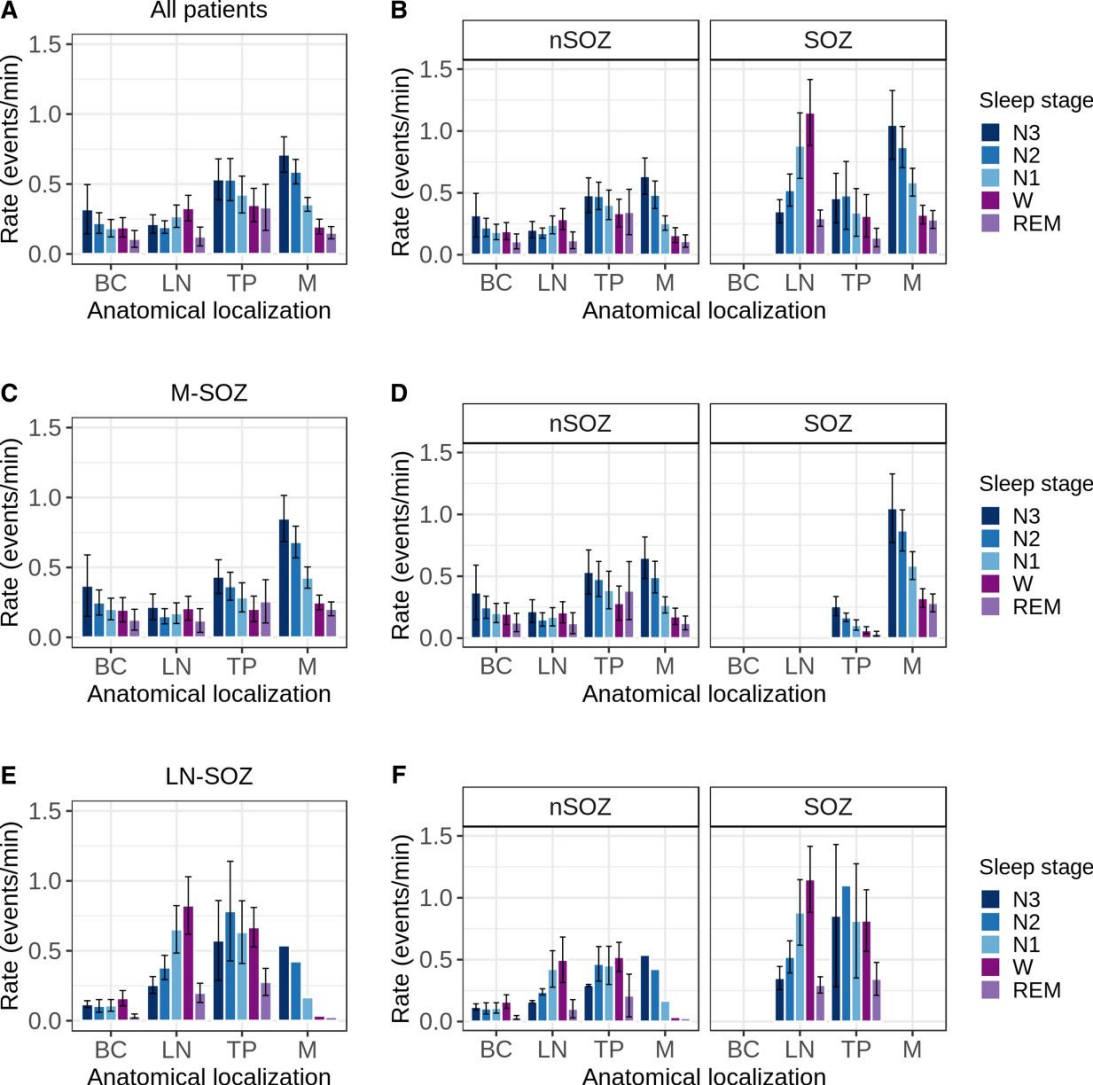
**Spatiotemporal distribution of IEDs across brain areas during wakefulness (W) and sleep (N1, N2, N3 non-REM and REM)**. (**A**) The rate of IEDs during wake and sleep in all patients. Wake and sleep conditioned the variability of IEDs within brain areas, with a mesial pattern in M areas, and LN pattern in LN areas. (**B**) The spatiotemporal distribution of the rate of IEDs in SOZ and non-SOZ in all patients. SOZ-associated brain areas showed a significant higher rate of IEDs compared with non-SOZ areas, with no detectable effect on the spatiotemporal distribution of IEDs across areas. REM sleep did not show significant variability across brain areas in both SOZ and non-SOZ (all *P* > 0.23). (**C**) In M-SOZ group, the IED rates across all brain areas differed significantly during the wake and sleep stages, and the variability was brain area dependent (*wake/sleep stages × brain areas interaction:* Wald *χ*^2^(2) = 9.3, *P* = 0.01). On M areas, a mesial pattern was found (Tukey’s *post hoc* tests; N2–N3 non-REM sleep versus Wake or REM sleep: *P* < 0.0001). Over TP, there was no significant difference between wake and sleep stages. (**D**) IED distribution in M-SOZ group patients across SOZ and non-SOZ electrode contacts: M areas showed a distinct mesial pattern. (**E**) IED distribution in LN-SOZ group. (**F**) IED distribution in LN group patients across SOZ and non-SOZ electrode contacts: LN showed a distinct LN pattern, with increased IED activity over SOZ-associated areas. BC, basal cortical; TP, temporal pole; M, mesial; LN, lateral neocortical; M-SOZ, mesial seizure onset zone; LN-SOZ, lateral neocortical seizure onset zone; LN pattern, lateral neocortical pattern.

The IEDs occurred broadly across the studied brain areas in all patients. Figure 3A represents the spatiotemporal distribution of the IEDs across brain areas during wakefulness and sleep stages in all patients.

When studying all patients, the stage of wakefulness and sleep, and the brain areas were found to significantly affect the rates of IED (*wake/sleep stages* *× brain areas interaction*; Wald *χ*^2^(df = 6) = 31.1, *P* < 0.0001).

During N2–N3 non-REM sleep, IED rates were significantly higher in M areas than in LN and BC areas (M versus LN: *P* < 0.0001; M versus BC: *P* = 0.0001) (Fig. 3A). However, there was no significant difference in IED rates between M and TP areas (*P* = 0.10). The IED rates were higher in TP areas than in LN areas (*P* = 0.012). We found no statistical differences in IED rates between brain areas during periods of wakefulness (all *P* > 0.11), and REM sleep (all *P* > 0.34).

Additionally, our analysis showed that the modulation of IEDs inside each brain area was conditioned by wake and sleep. M areas showed significantly higher IED rates during N2–N3 non-REM sleep than during wakefulness (*P* < 0.0001) and REM sleep (*P* < 0.0001). We refer to this pattern as the M pattern. In contrast, there was an inverse pattern seen in LN areas (referred to as the LN pattern), with significantly higher IED rates during wakefulness than during N2–N3 non-REM sleep (*P* = 0.04) and REM sleep (*P* = 0.010). We found no significant differences in IED rates across the wake and sleep stages in BC (all *P* > 0.40), and TP areas (all *P* > 0.06).

### SOZs influence IED rates across brain areas

SOZ affected IED rates differently depending on the anatomical location, regardless of wake or sleep stages (*SOZ* *× brain areas interaction:* Wald *χ*^2^(2) = 8.1, *P* = 0.02).

In Tukey’s *post hoc* analysis, the IED rates were significantly higher in SOZs than in non-SOZs in M areas (*P* = 0.002) and LN areas (*P* = 0.004), while there was no significant difference in TP areas (*P* = 0.94) (Fig. 3B and Supplementary Fig. 3).

We also found that IED rates in brain areas were affected by wake and sleep stages, regardless of their association with SOZ (*wake/sleep stages × brain areas interaction*: Wald *χ*^2^(4) = 29.9, *P* < 0.0001). During N2–N3 non-REM sleep in M areas, the rate of IEDs significantly increased compared with wakefulness and REM sleep (Tukey’s *post hoc* tests; N2–N3 versus Wake, and versus REM, *P* < 0.0001). While during wakefulness in LN areas, IEDs were significantly higher than REM sleep (*P* = 0.02), and to a lesser extent during N2–N3 non-REM sleep (*P* = 0.07).

Therefore, the quantitative effects of SOZ on IEDs are dependent on brain area, and the variability of IEDs within and across brain areas depends on the wake/sleep stage.

Because SOZ was not present in the same brain areas in all subjects, we analysed separately two groups of patients based on the anatomical location of SOZ. SOZ was present over M areas in M group, and it was present over LN areas (±TP) in the LN group.

#### The spatiotemporal distribution of IEDs differed in the M-SOZ and LN-SOZ patient groups

**Figure 4:**
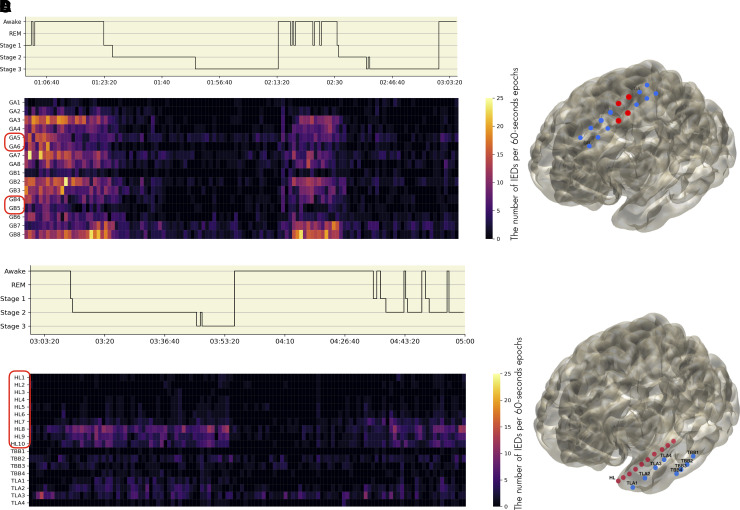
**Hypnograms and colour maps illustrating IED frequencies during wake and sleep in representative patients**. In the colour maps (**A and C**), lighter colours represent higher IED frequencies, whereas darker colours represent lower IED frequencies (**A and C**). SOZ are encircled in red. On the map of electrode contacts (**B and D**), SOZ are marked in red, whereas non-SOZ are shown in blue. (**A)** Hypnogram and colour map showing sleep staging and the spatial distribution of IEDs frequency in patient number 10 (LN-SOZ group). SOZ-involved areas were recorded by electrode contacts GA5, GA6 and GB4,5. According to colour changes, the frequency of IEDs is higher during wakefulness than during N2, and N3 non-REM sleep (LN pattern). (**B)** Grid electrode placed over the left lateral frontal and parietal areas (not entirely shown) in patient number 10, showing SOZs (GA5, GA6 and GB4,5) and non-SOZs contacts. (**C**). Hypnogram and colour map showing sleep staging and the spatial distribution of IEDs frequency in patient number 4 (M-SOZ group). SOZs in left TP and amygdalo-hippocampal areas were covered by depth electrodes ‘HL1–HL10’. The colour changes show higher IED frequencies in M areas involved during N2, and N3 non-REM sleep, as well as lower frequencies during wakefulness (Mesial pattern). In contrast, a noticeable IED activity on the TLA3 (lateral temporal cortex) occurred mainly during periods of wakefulness. (**D**) Virtual brain showing the anatomical location of electrode contacts for patient 4. Depth electrode contacts ‘HL1–HL10’ in the left TP and amygdalo-hippocampal areas. TLA1 was placed on the left TP. TLA3 and TLA 4 were placed on the left lateral temporal cortex, and the TBB over the left basal temporal areas. SOZs, seizure onset zones; M-SOZ, mesial seizure onset zone; LN-SOZ, lateral neocortical seizure onset zone.

The spatiotemporal distribution of IED rates in the M-SOZ and LN-SOZ groups is shown in Fig. 3C and D and in Fig. 3E and F, respectively. For each group, Fig. 4 displays examples of the variability in IED rates during the wake and sleep stages.

#### M-SOZ group

The results showed that SOZ in the TP and M areas had a significant effect on the rate of IEDs and the effect was dependent on the brain area (*brain area × SOZ interaction:* Wald *χ*^2^ (1) = 14.0, *P* = 0.0002, Fig. 3D).

The IED rates in SOZs were significantly higher than those non-SOZs in M areas (*P* = 0.003). In TP areas, however, this effect was not evident, as IED rates were higher in non-SOZs than in SOZs (*P* = 0.08).

The mesial pattern appears to be independent of SOZ in the M-SOZ group. It is, rather, a characteristic of the brain area modulated by wake and sleep. SOZ was found to have a quantitative effect on the rate of IEDs.

#### LN-SOZ group

In the LN-SOZ group, the significant influence of wakefulness and sleep stages, as well as SOZ on IEDs rates was independent of anatomical location (*wake/sleep stages:* Wald *χ*^2^ (2) = 13.0, *P* = 0.001, *SOZ*: Wald *χ*^2^(1) = 13.0, *P* = 0.0003).

We found that the rate of IEDs was higher during wakefulness than during REM sleep (Tukey’s *post hoc* test; *Wake/REM sleep*: *P* = 0.008). Furthermore, SOZ-included areas had a significantly higher rate of IEDs than non-SOZ-included areas (SOZ/non-SOZ: *P* = 0.004).

### The morphological features vary across brain areas

The percentage of data points that were excluded ranged from 0 to 1.8%. Supplementary Table 3 presents the distribution of values extracted from the iEEG data for each morphological parameter (min, max, first quartile, median, third quartile, interquartile range and outliers).

**Figure 5:**
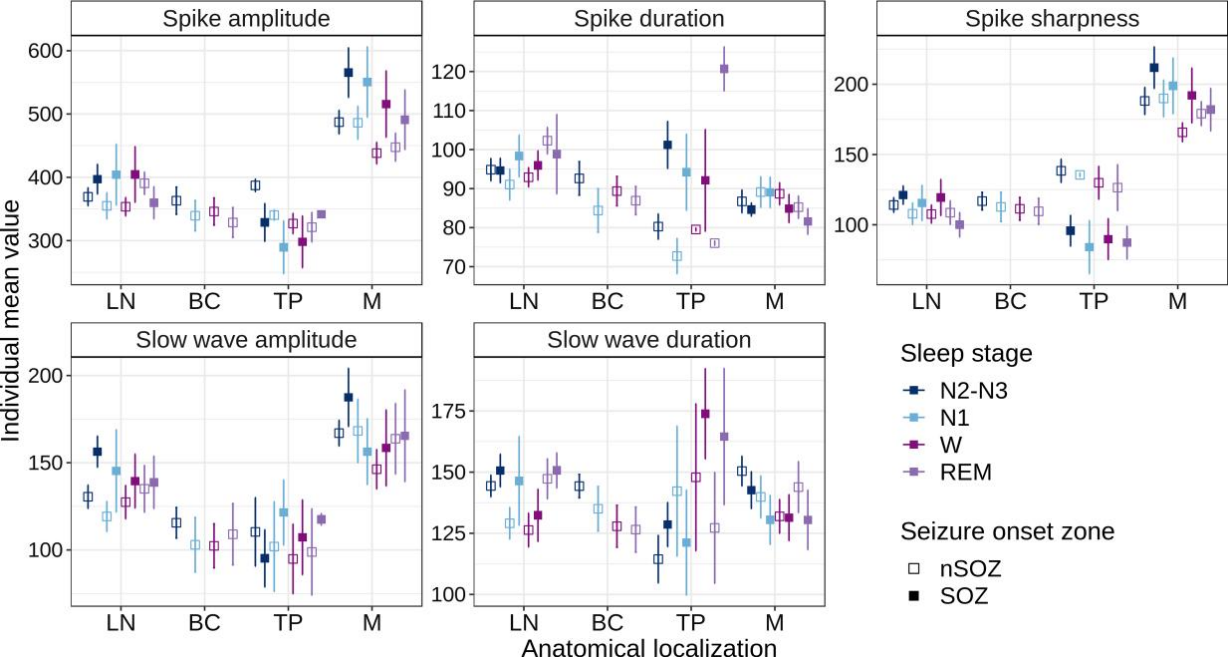
**Morphological characteristics of IEDs during wake and sleep across brain areas, as well as in SOZs and non-SOZs**. Each morphological characteristic of IEDs (amplitude, sharpness and duration of spikes and slow waves) is illustrated during wake and sleep stages (N2–N3 combined), as well as across brain areas. The variation of the characteristics based on affiliation to SOZ is shown. Spike amplitudes in M areas were significantly higher than in LN and TP areas (Tukey’s *post hoc*; M > LN and M > TP: *P* < 0.0001). Based on the estimated differences, these changes were maintained during wake and sleep stages (*Wake/Sleep stages*: Wald *χ*^2^(3) = 4.4, *P* = 0.2, interaction *area × Wake/Sleep stages*: (6) = 3.6, *P* = 0.7). Spikes in M areas were significantly sharper than those in LN and TP areas (Tukey’s *post hoc*; M > LN: *P* = 0.0009, M > TP: *P* < 0.0001). Compared with non-SOZ areas, SOZ showed an effect on spike sharpness in M and TP areas, but not in LN-areas. In M areas, SOZ spikes were significantly sharper than non-SOZ spikes. In TP areas, this effect was reversed, as SOZ suppressed spike sharpness relative to non-SOZ (M SOZ > non-SOZ: *P* = 0.03, TP non-SOZ > SOZ: *P* < 0.0001). Slow-wave amplitudes were higher in M areas than in LN and TP areas (M > LN and M > TP: *P* < 0.0001). LN areas displayed significantly longer spike duration than M areas (Tukey’s *post hoc*; *N2–N3 non-sleep*: *P* = 0.009, *REM sleep: P* = 0.05, and *wake: P* = 0.0002). SOZ associated with LN and TP areas had significantly longer spike durations than SOZ associated with M areas (SOZ *M* < SOZ *LN*, and SOZ *M* < SOZ *TP*: *P* < 0.0001). During wakefulness, the slow-wave durations were significantly longer in TP areas than in LN areas or in M areas (Tukey’s *post hoc*; TP/LN: *P* = 0.04; TP/M: *P* = 0.02). There was a significant difference only in SOZ- involved areas, with LN and TP areas having significantly longer slow-wave durations than M areas (Tukey’s *post hoc*; *SOZ* LN > M: *P* = 0.03; *SOZ* TP > M: *P* = 0.02). There were no variations in non-SOZ areas. The amplitudes are expressed in microvolt (μV) and the durations in milliseconds. BC, basal cortical; TP, temporal pole; M, mesial; LN, lateral neocortical; SOZs, seizure onset zones.

Figure 5 displays the morphological characteristics of IEDs in different brain areas during wake and sleep (N2–N3 combined), as well as in SOZs and non-SOZs, and Supplementary Fig.4 depicts N2, and N3 separately.

Spike amplitudes, and sharpness, as well as slow-wave amplitudes differed according to their anatomical location (*spike amplitude*: Wald *χ*^2^(3) = 48.0, *P* < 0.0001; *spike sharpness:* Wald *χ*^2^(3) = 69.4, *P* < 0.0001; *slow-wave amplitude*: Wald *χ*^2^(3) = 24.8, *P* = 0.0001).

Spikes in M areas had greater amplitudes and sharper slopes than spikes in other areas (Tukey’s *post hoc*; *spike amplitudes*: M > LN, *P* = 0.0004, M > BC, *P* < 0.0001 and M > TP *P* = 0.0002; *sharpness*: M > LN, M > BC and M > TP, *P* < 0.0001). Slow-wave amplitudes in M areas were significantly higher than in BC and TP areas (M > BC: *P* = 0.001, M > TP: *P* = 0.004) but did not differ significantly from those in LN areas (M/LN: *P* = 0.1).

### SOZs modulate the morphological features

*Spike amplitudes* differed according to anatomical location (M, LN, TP areas) and SOZs affiliation [*brain areas*: Wald *χ*^2^(2) = 90.9, *P* < 0.0001, *SOZ*: Wald *χ*^2^(1) = 5.5, *P* = 0.02; *brain area × SOZ interaction*: Wald *χ*^2^(2) = 3.2, *P* = 0.2]. Figure 5 illustrates the differences in spike amplitudes between brain areas. We found that spikes amplitudes associated with SOZ areas were higher than those associated to non-SOZ area (*SOZ/non-SOZ*: *P* = 0.02).

*Spike Sharpness* varied by anatomical location, and the influence of SOZs was found to be brain area dependent (*brain areas × SOZ interaction:* Wald *χ*^2^(2) = 21.3, *P* < 0.0001).

*Spike sharpness* appeared to be a brain area-dependent attribute, with spikes in M areas being sharper during wake and sleep than spikes in other areas, and the effect of SOZ was also conditioned by brain area. Figure 5 shows how spike sharpness differs between brain areas and how SOZ affects them.

We found that s*low-wave amplitudes* vary depending on anatomical location, with no statistically significant effect for SOZ [*brain area*: Wald *χ*^2^(2) = 29.6, *P* < 0.0001] (Fig. 5).

Within each area, slow-wave amplitudes in SOZ and non-SOZ did not differ significantly (*LN* non-SOZ/SOZ: *P* = 0.13; *M* non-SOZ/SOZ: *P* = 0.7; *TP* non-SOZ/SOZ: *P* = 0.69).

According to these results, the amplitudes of spikes and slow waves, as well as the sharpness of spikes, are brain area attributes that are independent of wake and sleep. Further, the effects of SOZ were dependent on anatomical location.

The brain area was found to condition the influence of wakefulness and sleep on spike duration (*brain areas × wake/sleep stages interaction:* Wald *χ*^2^(6) = 11.9, *P* = 0.06). The spatial distribution of spike durations is illustrated in Fig. 5.

*Spike duration* varied across brain areas, and the variability was also SOZ dependent (*brain areas:* Wald *χ*^2^(2) = 18.3, *P* < 0.0001, *brain areas × SOZ interaction:* Wald *χ*^2^(2) = 39.6, *P* < 0.0001).

SOZ had a significant influence on the M and TP areas. In M areas, spikes associated with SOZ had a shorter duration than non-SOZ spikes (Tukey’s *post hoc*; *M* SOZ < non-SOZ *P* = 0.05). While in TP areas, spikes associated with SOZ showed longer spike durations than non-SOZ spikes (*TP* SOZ > non-SOZ *P* < 0.0001).

The duration of slow waves varied by anatomical location and wake/sleep stage (*brain areas × wake/sleep stages interaction*: Wald *χ*^2^(6) = 15.1, *P* = 0.02), and the effects of SOZ were found to be brain area dependent (*brain areas × SOZ interaction:* Wald *χ*^2^(2) = 4.2, *P* = 0.02). Figure 5 shows the variability of slow-wave durations across brain areas and the effects of SOZs.

Based on these findings, spike and slow-wave durations were modulated by wake and sleep across brain areas and locally by SOZ.

After statistical adjustment for electrode type, the results remained consistent. Supplementary Statistical Analysis File shows the details of the statistical tests.

## Discussion

Our results suggest that wake and sleep stages, as well as SOZs, influence the spatial distribution and the morphological characteristics of IEDs.

### The spatiotemporal distribution patterns of IEDs are location- and sleep-dependent

In our study, we discovered that variability in IEDs rates depended both on their anatomical locations and their wake and sleep states. In previous studies, non-REM sleep was found to promote IEDs in focal refractory temporal epilepsy patients monitored with scalp EEG,^6,28^ and invasive recordings.^29^ Del Felice *et al*.^15^ also found that scalp EEG during N2 non-REM sleep in right temporal lobe epilepsy showed a broader distribution of IEDs than during wakefulness.

Nevertheless, another pattern of IEDs distribution was observed in which IEDs were more correlated with wakefulness and REM sleep than non-REM sleep. In a group of patients with focal temporal epilepsy, with focal seizures and impaired consciousness, the maximum spiking rates occurred in one patient during wakefulness and in five patients during REM sleep.^28^

We did not find a statistically significant difference between wakefulness and sleep in our study, although it was heavily weighted towards N2 and N3 sleep (Fig. 2A). The reason may be due to increased IEDs activity during wakefulness in LN-SOZ, which reduced the gap between wakefulness and sleep. There is a possibility that the observed difference between studies regarding the rate of IED activity during wakefulness and sleep may be explained by a difference in the number of patients with SOZ located in the mesial or lateral neocortex.

To identify the mechanisms behind this strong association between IEDs, wakefulness and sleep, earlier studies demonstrated a functional interaction between IEDs and sleep oscillatory activities.^30–32^

### SOZs are associated with higher IED rates in LN and M areas

The relationship between IEDs and seizures is still unclear. The lack of temporality between interictal spikes and seizures,^33^ led to the assumption that IEDs and seizures were independent events.^34^ In the present study, we found that SOZs enhanced the rate of IEDs in M and LN areas as well as the spatiotemporal distribution patterns of IEDs. This is in agreement with Del Felice *et al*.,^15^ therefore, IEDs and SOZs are likely linked by an underlying common mechanism, which is possibly regulated by wake and sleep.

In contrast to a previous report,^35^ we found that SOZs have a differential influence on the rate of IEDs during wakefulness and sleep, depending on the brain area. Although SOZs had higher IED rates than non-SOZs in the M areas, they shared similar variability during wakefulness and sleep (mesial pattern).

During wakefulness, in LN areas, SOZs were associated with higher IED rates, with SOZs having higher IED rates than in non-SOZs (LN pattern). Our results are consistent with previous studies,^15,29^ leading us to speculate that the increase in IEDs during non-REM sleep in M areas, and during wakefulness in LN areas, reflects a local modulatory mechanism connected to the oscillatory properties of the vigilance states.

There is a controversy on the value of different sleep stages in localizing epileptic activity.^36–39^ Overall, our findings indicate that REM sleep has a lower IEDs rates than other sleep stages. Our results are consistent with a previous work.^40^ The mechanism underlying this suppressive effect may be due to cortical desynchronization, presumably related to the nature of REM sleep.

Based on our findings, we hypothesize a connection between IEDs and neuronal activity through wakefulness- and sleep-related oscillatory mechanisms, which operate, at least in part, through synchronization and desynchronization.

### The M-SOZ and LN-SOZ patient groups displayed opposing IED spatiotemporal distribution patterns

Compared with non-SOZ groups, the presence of SOZs in M-SOZ and LN-SOZ groups did not change the pattern of variability of IEDs, but rather facilitated IEDs. According to this finding, SOZs can be considered as local zones where spontaneously produced wake and sleep-related neuronal activities are altered. This alteration allows high expression of IEDs within SOZ-associated with M areas (mainly during non-REM sleep), with LN areas (mainly during wakefulness) and lower rates of IEDs in SOZs TP areas in the M-SOZs group. It is possible that these changes are provoked by local vigilance state or a transformation of sleep-related physiological activity (replacing an ongoing physiological activity by interictal discharges). This is in agreement with Frauscher *et al*.,^41^ who reported that the presence of hippocampal spiking lowered the rate of hippocampal spindles. Identifying these patterns in patients with mesial and LN SOZs can improve our understanding of cognitive decline and guide selective surgery.

We found that SOZs suppressed IEDs in the TP areas. It raises questions about the specificity and the nature of the sleep oscillations in TP areas. Regarding IEDs activity, the impact of sleep seems to be determined by sleep-related oscillatory properties and their synchronizing mechanisms. This observation was confirmed earlier by Ferrillo *et al*.^42^ According to Frauscher *et al*.^40^ and Campana *et al*.,^43^ phasic REM sleep suppressed IEDs more than tonic REM sleep. Ferrillo *et al*.^42^ attributed the less occurrence of IEDs during REM sleep to the presence of synchronized theta activity.

### Regional IED morphological characteristics are consistent across wake and sleep stages and modulated by SOZs

Although previous studies have presumed that spike amplitudes increase during non-REM sleep and decrease during REM sleep,^44^ to the best of our knowledge, the present study is the first to analyse the morphological characteristics of IEDs across broad brain areas, during wakefulness and sleep stages, as well as investigate the effect of SOZs.

We found that the morphological characteristics of the IEDs were modulated differently during wakefulness and sleep across brain areas. However, they remained consistent within restricted brain areas. This pattern of modulation is most likely produced by sleep-related oscillatory activity, which orchestrates wakefulness and sleep across brain areas. According to Wendling *et al*.,^45^ the spatial distribution and the level of synchronization of pyramidal cells are critical factors that influence the morphology of epileptic events. Our findings suggest that SOZs influence the interictal configurations through local neuronal modulatory mechanisms, which may reflect a distinct vigilance state. This finding confirms a previous report by Ferrillo *et al*.^42^

We believe that both interictal discharges and seizures originate from sleep-related oscillatory activity depending on anatomical location. Revealing the underlying local vigilance states of SOZs will allow us to gain a better understanding of disease pathophysiology and improve targeting therapies.

## Conclusion

By analysing big data from iEEG using automatic detection methods, we demonstrate the modulation of IEDs rates and their morphological features by naturally occurring wakefulness and sleep across four large-scale brain areas. According to our findings, the wake and sleep stages, together with SOZs affiliations, contribute to the generation of predictable patterns of IED activity. This research emphasizes the importance of using a wider temporal analysis to view and determine IED dynamics. Analysing the variability of IED rates and the morphological characteristics could provide a potential method for identifying SOZ-involved brain areas, and provide a finer interpretation of the interictal network, while considering the local and global sleep-related oscillatory mechanisms.

## Limitation

The small sample size of the LN-SOZs group, restricted by the selection based on the inclusion criteria, limited the results although they were rigorously conducted. Accordingly, the results should be interpreted with caution and need to be reproduced using a larger sample size. Nevertheless, we found that the analysis of this group will contribute to a better understanding of IED dynamics and provide a starting point for future research.

Due to the complexity of analysing multiple conditions and factors, we decided not to include spike source analysis in our comprehensive assessment of IEDs.

## Supplementary Material

fcac183_Supplementary_Data

## Abbreviations

BC =: basal cortical
CD =: cortical dysplasia
HS =: hippocampal sclerosis
HSD =: honestly significant difference
IED =: interictal epileptiform discharge
iEEG =: intracranial EEG
LN =: lateral neocortical
M =: mesial temporal
REM =: rapid eye movement
SOZ =: seizure onset zone
TP =: temporal pole.
