# Supplementary material for: Interictal epileptiform discharges show distinct spatiotemporal and morphological patterns across wake and sleep
Source: Brain Commun. 2022 Jul 18;4(5):fcac183. doi: 10.1093/braincomms/fcac183 (PMC9724782; doi:10.1093/braincomms/fcac183)
Supplement: fcac183_Supplementary_Data [file fcac183_Supplementary_Data.zip › Supplementary Figures and legends-18082022.pdf]

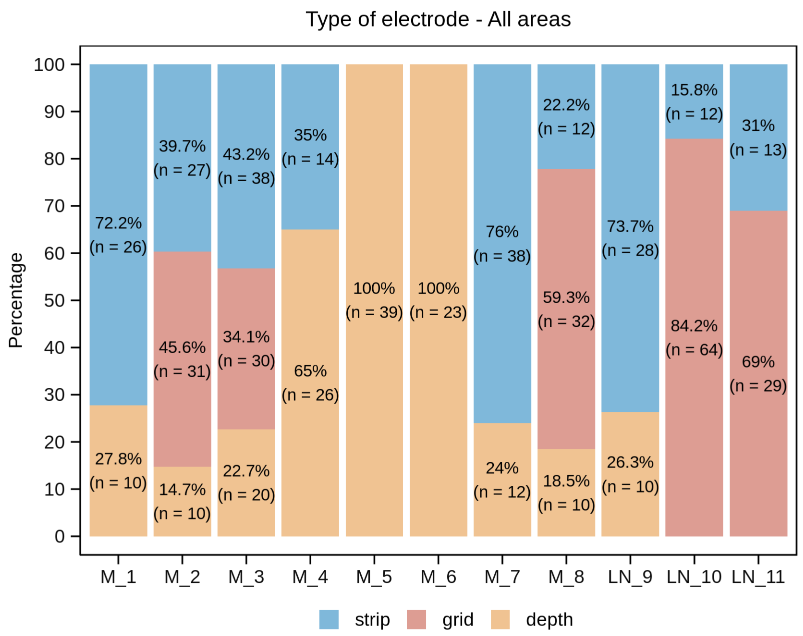

Supplementary Fig. 1: The distribution of the type of electrode used for recording intracranial EEG in all brain areas studied, by patient.
For intracranial recording, three different types of electrodes were employed. Each type is represented by a distinct color. The absolute number of electrode contacts is indicated between brackets. Each patient's percentage of electrode type representation is shown above the absolute number.
The distribution of the type of electrode in all brain areas studied, by patient: Depth electrodes were the main type in M-SOZ patients' numbers 4, 5, and 6, whereas grid electrodes were the main type in LN-SOZ patients' numbers 10 and 11.

M = patient in the M-SOZ group, followed by the number of the patient.
LN = patient in LN-SOZ, followed by the number of the patient.
M-SOZ : Mesial seizure onset zone.
LN-SOZ: Lateral neocortical seizure onset zone.

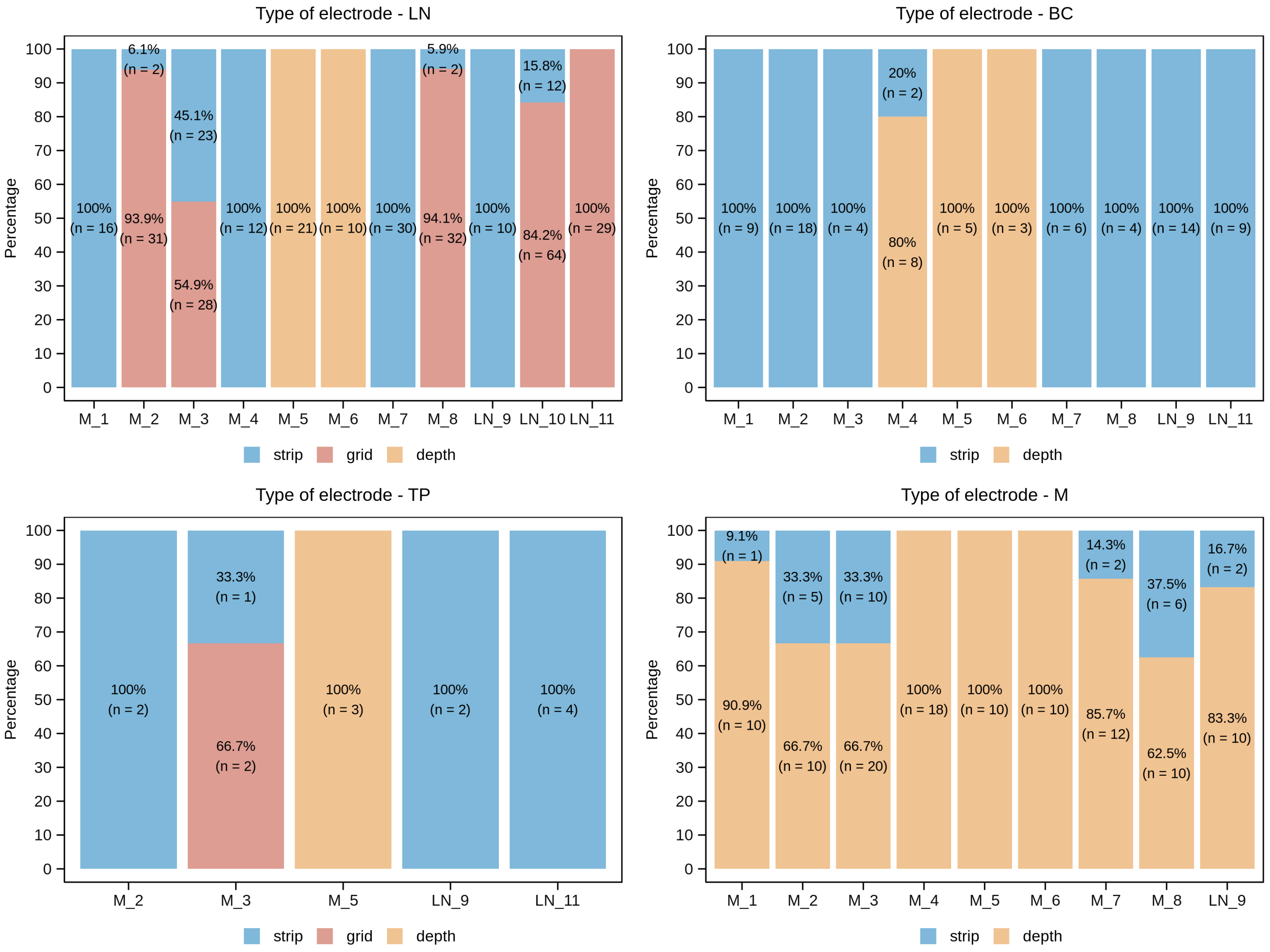

Supplementary Fig. 2: The distribution of the type of electrode used for recording intracranial EEG by brain area, and by patient.

The distribution of the type of electrode by brain area studied, and by patient: M areas were mainly explored by depth electrodes, while LN areas were mainly recorded by grid and strip electrodes.

M = patient in the M-SOZ group, followed by the number of the patient.

LN = patient in LN-SOZ, followed by the number of the patient.

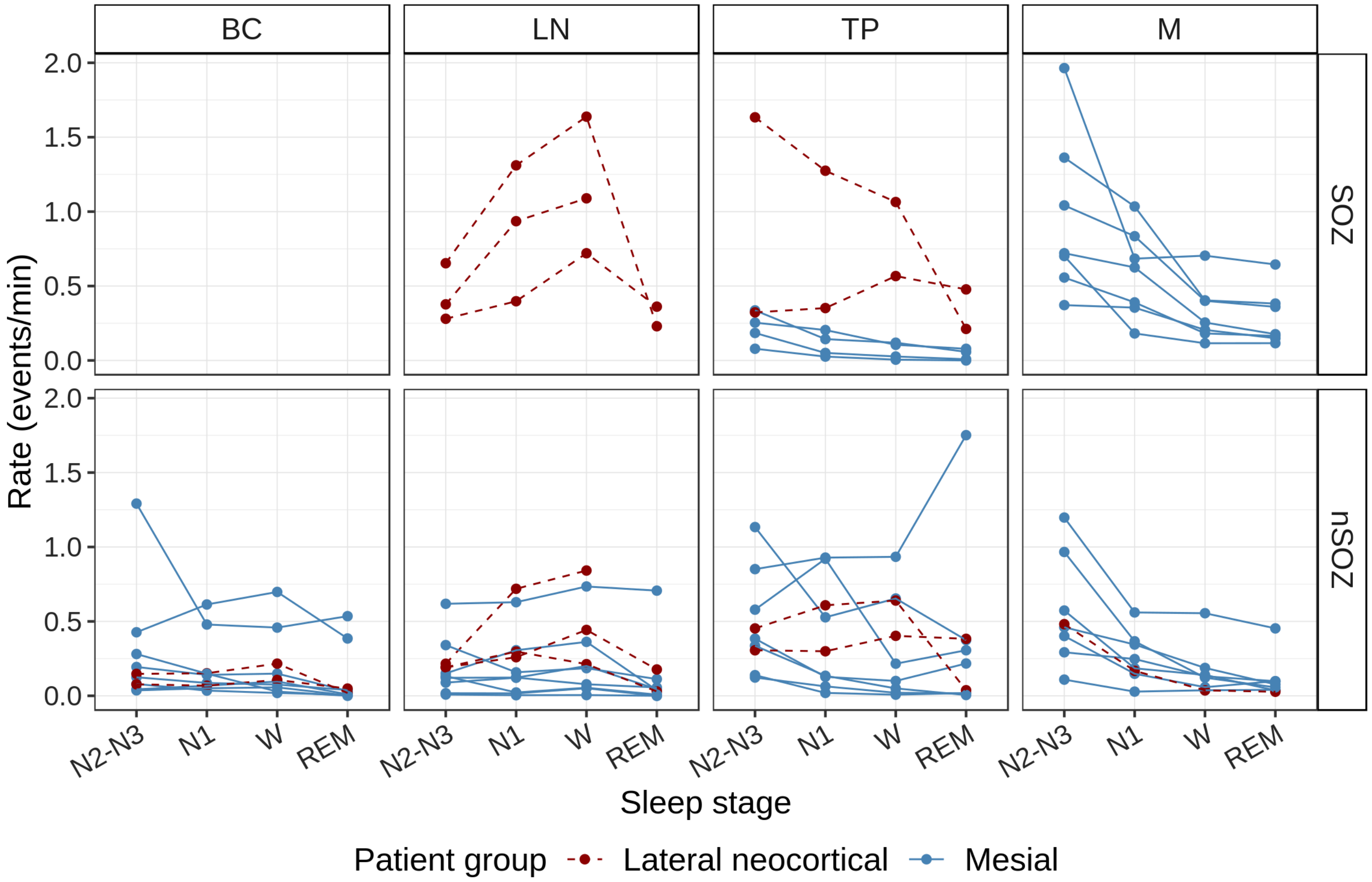

Supplementary Fig. 3: IED rate distribution in the two patient groups during wakefulness (W) and the different sleep stages (N1, N2, N3 non-REM, and REM) across the four studied brain areas, and according to SOZ and non-SOZ electrode contacts.
IED distribution in patients' groups and in SOZ versus non-SOZ.

BC: Basal cortical, TP: Temporal pole, M: Mesial, LN: Lateral neocortical.
SOZ: Seizure onset zone.
M-SOZ : Mesial seizure onset zone.
LN-SOZ: Lateral neocortical seizure onset zone.

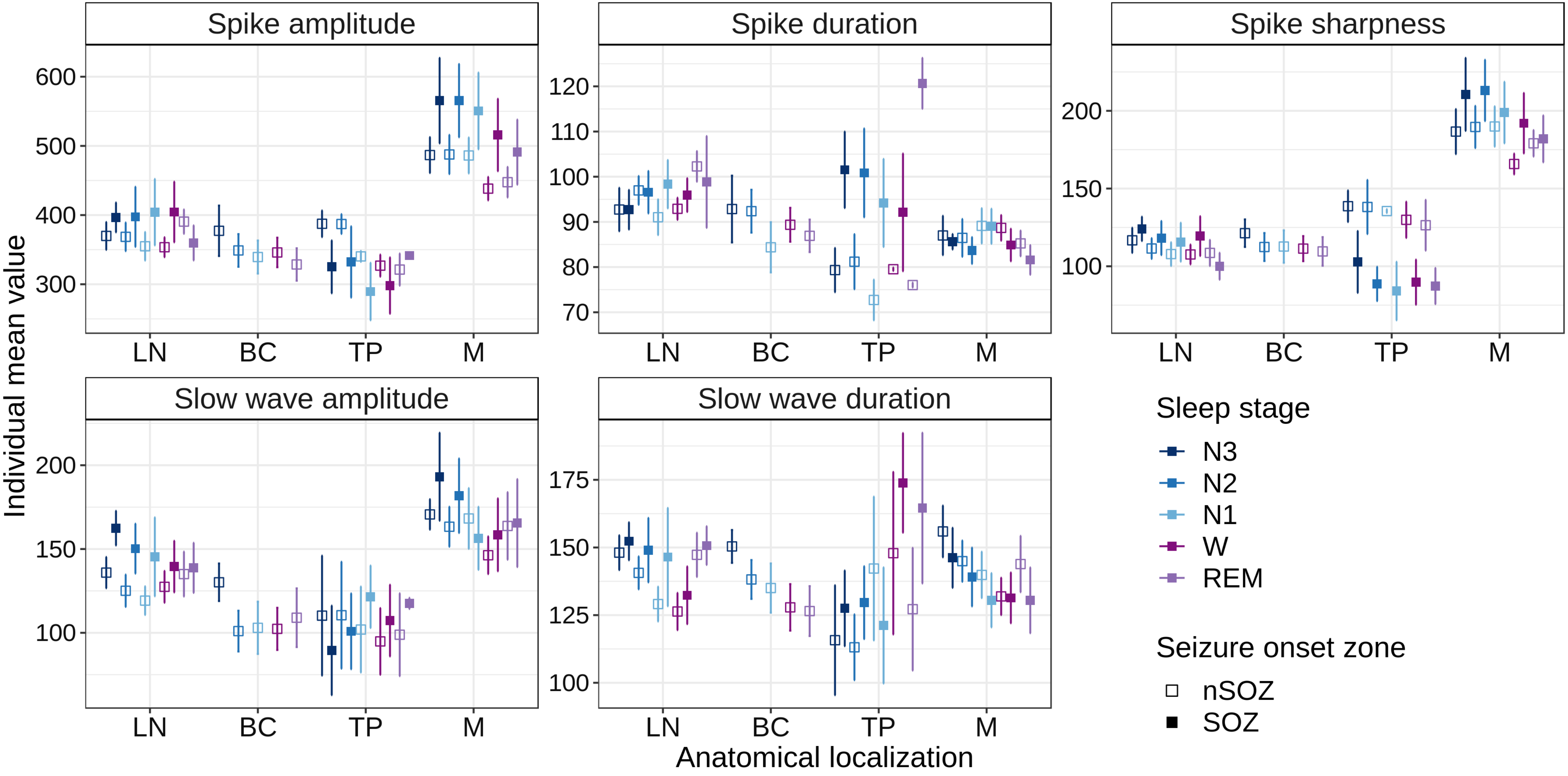

Supplementary Fig. 4: Morphological characteristics of IEDs during wake and sleep across brain areas, as well as in SOZs and non-SOZs (N2 and N3 separated).
Each morphological characteristic of IEDs (amplitude, sharpness, and duration of spikes and slow waves) is illustrated during wake and sleep stages (N2-N3 separated), as well as across brain areas. The variation of the characteristics based on affiliation to SOZ is shown.
The amplitudes are expressed in microvolt (µV) and the durations in milliseconds.
N2 and N3 show almost the same changes across brain areas.
