# Supplementary material for: Interictal epileptiform discharges show distinct spatiotemporal and morphological patterns across wake and sleep
Source: Brain Commun. 2022 Jul 18;4(5):fcac183. doi: 10.1093/braincomms/fcac183 (PMC9724782; doi:10.1093/braincomms/fcac183)
Supplement: fcac183_Supplementary_Data [file fcac183_Supplementary_Data.zip › Supplementary Statistical Analysis.pdf]

Interictal epileptiform discharges show distinct spatiotemporal and morphological patterns across wake and sleep

Statistical analysis

Amal Fouad, Hamed Azizollahi, Jean-Eudes Le Douget, François-Xavier Lejeune, Mario Valderrama, Liliana Mayor, Vincent Navarro, Michel Le Van Quyen

Contents

1 Spike rates according to SOZ (one model per patient group LN or M) 2
1.1 All subjects (M, LN and TP areas) 2
1.2 M-SOZ group 4
1.3 LN-SOZ group 6

2 Morphological differences between areas (no adjustment for the type of electrode) 8

3 Morphological parameters (N2 and N3 combined, no adjustment for electrode type) 9
3.1 Spike amplitude 9
3.2 Spike sharpness 10
3.3 Slow wave amplitude 12
3.4 Spike duration 13
3.5 Slow wave duration 15

4 Morphological parameters (N2 and N3 combined) (with adjustment for electrode type) 17
4.1 Spike amplitude 17
4.2 Spike duration 18
4.3 Spike sharpness 19
4.4 Slow wave amplitude 20
4.5 Slow wave duration 21

5 R session information 23

1 Spike rates according to SOZ (one model per patient group LN or M)

1.1 All subjects (M, LN and TP areas)

1.1.1 Linear mixed model with Type II Wald chi-square tests

Table 1: LMM (Type II Wald chi-square tests) with Sleep, Area and SOZ

	Chisq	Df	Pr(>Chisq)
Sleep	21.90281	2	0.0000175
Area	12.64297	2	0.0017973
Sleep:Area	26.91405	4	0.0000207

1.1.2 Tukey's post hoc comparisons

Table 2: Post hoc Tukey's pairwise comparisons of brain areas per wake/sleep stage

contrast	Sleep	estimate	SE	df	t.ratio	p.value
LN - M	N2-N3	-0.5375150	0.0918326	161.4980	-5.8532047	0.0000001
LN - TP	N2-N3	-0.2826163	0.0903056	159.8813	-3.1295531	0.0058705
M - TP	N2-N3	0.2548988	0.0883627	157.5048	2.8846856	0.0123549
LN - M	REM	-0.0578698	0.1312906	156.3553	-0.4407766	0.8985286
LN - TP	REM	-0.1463250	0.1301883	155.2854	-1.1239492	0.5008007
M - TP	REM	-0.0884552	0.1237414	156.3421	-0.7148388	0.7550920
LN - M	W	0.2042894	0.1272996	158.7588	1.6047923	0.2465936
LN - TP	W	0.0948242	0.1262025	157.6936	0.7513656	0.7332492
M - TP	W	-0.1094652	0.1237414	156.3421	-0.8846287	0.6507964

Table 3: Post hoc Tukey's pairwise comparisons of wake/sleep stages per area

contrast	Area	estimate	SE	df	t.ratio	p.value
N2-N3 - REM	LN	0.0751176	0.1171359	157.8105	0.6412860	0.7975504
N2-N3 - W	LN	-0.2306261	0.1098177	155.0636	-2.1000808	0.0931248
REM - W	LN	-0.3057437	0.1331946	157.2195	-2.2954670	0.0593561
N2-N3 - REM	M	0.5547628	0.1060940	155.0636	5.2289732	0.0000016
N2-N3 - W	M	0.5111783	0.1060940	155.0636	4.8181630	0.0000102
REM - W	M	-0.0435845	0.1225068	155.0636	-0.3557720	0.9326423
N2-N3 - REM	TP	0.2114089	0.1060940	155.0636	1.9926559	0.1174086
N2-N3 - W	TP	0.1468143	0.1060940	155.0636	1.3838135	0.3518579
REM - W	TP	-0.0645945	0.1225068	155.0636	-0.5272731	0.8580981

1.2 M-SOZ group

1.2.1 Linear mixed model with Type II Wald chi-square tests

Table 4: LMM (Type II Wald chi-square tests) with Sleep, Area and SOZ

	Chisq	Df	Pr(>Chisq)
Sleep	42.326095	2	0.0000000
Area	2.920193	1	0.0874774
SOZ	3.152354	1	0.0758175
Sleep:Area	9.251779	2	0.0097949
Sleep:SOZ	1.320337	2	0.5167643
Area:SOZ	13.994058	1	0.0001834
Sleep:Area:SOZ	1.221924	2	0.5428284

1.2.2 Tukey's post hoc comparisons

Table 5: Post hoc Tukey's pairwise comparisons SOZ-nSOZ per area

contrast	Area	estimate	SE	df	t.ratio	p.value
nSOZ - SOZ	M	-0.2602547	0.0837186	82.14081	-3.108684	0.0025821
nSOZ - SOZ	TP	0.1815918	0.1040363	84.29451	1.745465	0.0845478

Table 6: Post hoc Tukey's pairwise comparisons of areas per SOZ

contrast	SOZ	estimate	SE	df	t.ratio	p.value
M - TP	nSOZ	-0.1286326	0.0837186	82.14081	-1.536488	0.1282620
M - TP	SOZ	0.3132139	0.1040363	84.29451	3.010621	0.0034393

Table 7: Post hoc Tukey's pairwise comparisons of wake/sleep stages per area

contrast	Area	estimate	SE	df	t.ratio	p.value
N2-N3 - REM	M	0.5619431	0.0946769	81.00498	5.9353770	0.0000002
N2-N3 - W	M	0.5159096	0.0946769	81.00498	5.4491611	0.0000016
REM - W	M	-0.0460334	0.1093235	81.00498	-0.4210753	0.9070134
N2-N3 - REM	TP	0.1498529	0.1110185	81.00498	1.3498016	0.3721116
N2-N3 - W	TP	0.1864129	0.1110185	81.00498	1.6791154	0.2194002
REM - W	TP	0.0365599	0.1281931	81.00498	0.2851942	0.9561791

Table 8: Post hoc Tukey's pairwise comparisons of areas per wake/sleep stage

contrast	Sleep	estimate	SE	df	t.ratio	p.value
M - TP	N2-N3	0.3394862	0.0863692	81.96680	3.9306398	0.0001762
M - TP	REM	-0.0726039	0.1206479	81.50377	-0.6017832	0.5489888
M - TP	W	0.0099895	0.1206479	81.50377	0.0827985	0.9342148

1.3 LN-SOZ group

1.3.1 Linear mixed model with Type II Wald chi-square tests

Table 9: LMM (Type II Wald chi-square tests) with Sleep, Area and SOZ

	Chisq	Df	Pr(>Chisq)
Sleep	13.0313280	2	0.0014801
Area	2.4530080	1	0.1172999
SOZ	12.9973309	1	0.0003119
Sleep:Area	4.4953948	2	0.1056422
Sleep:SOZ	1.4028124	2	0.4958875
Area:SOZ	0.1604188	1	0.6887712
Sleep:Area:SOZ	2.1981201	2	0.3331841

1.3.2 Tukey's post hoc comparisons

Table 10: Post hoc Tukey's pairwise comparisons SOZ-nSOZ

contrast	estimate	SE	df	t.ratio	p.value
nSOZ - SOZ	-0.3512882	0.1115605	24.07546	-3.148858	0.0043351

Table 11: Post hoc Tukey's pairwise comparisons of wake/sleep stages

contrast	estimate	SE	df	t.ratio	p.value
N2-N3 - REM	0.2562142	0.1358163	24.77143	1.886476	0.1636589
N2-N3 - W	-0.2479326	0.1247284	24.07546	-1.987779	0.1368543
REM - W	-0.5041468	0.1537264	24.62662	-3.279508	0.0083906

Table 12: Post hoc Tukey's pairwise comparisons of wake/sleep stages per area

contrast	Area	estimate	SE	df	t.ratio	p.value
N2-N3 - REM	LN	0.1111167	0.1909111	25.36895	0.5820340	0.8308807
N2-N3 - W	LN	-0.5059821	0.1577704	24.07546	-3.2070792	0.0101451
REM - W	LN	-0.6170988	0.2115282	25.16675	-2.9173354	0.0193746
N2-N3 - REM	TP	0.4013116	0.1932285	24.07546	2.0768763	0.1159438
N2-N3 - W	TP	0.0101169	0.1932285	24.07546	0.0523571	0.9984899
REM - W	TP	-0.3911947	0.2231210	24.07546	-1.7532851	0.2066333

Table 13: Post hoc Tukey's pairwise comparisons of areas per wake/sleep stage

contrast	Sleep	estimate	SE	df	t.ratio	p.value
LN - TP	N2-N3	-0.3682940	0.1549235	25.80405	-2.3772639	0.0251456
LN - TP	REM	-0.0780991	0.2231210	24.07546	-0.3500303	0.7293597
LN - TP	W	0.1478050	0.2115282	25.16675	0.6987481	0.4911169

2 Morphological differences between areas (no adjustment for the type of electrode)

Table 14: LMM (Type II Wald chi-square tests)

Parameter	Chisq	DF	Pvalue	padj
Spike amplitude	48.007	3	0.000000	0.000000
Spike duration	6.942	3	0.073756	0.516292
Spike sharpness	69.377	3	0.000000	0.000000
Slowwave amplitude	24.764	3	0.000017	0.000119
Slowwave duration	2.871	3	0.411881	1.000000

```
## Tukey's post hoc comparisons: Spike amplitude
## ----------------------------------------------------
##  contrast estimate   SE   df t.ratio p.value
##  LN - BC     37.55 24.8 22.0  1.511  0.4480
##  LN - TP     45.53 32.0 23.7  1.423  0.4982
##  LN - M    -125.01 25.7 22.3 -4.857  0.0004
##  BC - TP      7.98 32.3 23.2  0.247  0.9945
##  BC - M    -162.57 26.1 21.6 -6.240  <.0001
##  TP - M    -170.54 33.3 23.8 -5.121  0.0002
##
## P value adjustment: tukey method for comparing a family of 4 estimates
## Tukey's post hoc comparisons: Spike sharpness
## ----------------------------------------------------
##  contrast estimate   SE   df t.ratio p.value
##  LN - BC      3.93 10.8 22.1  0.364  0.9831
##  LN - TP      7.51 13.9 24.9  0.542  0.9478
##  LN - M     -76.88 11.2 22.6 -6.867  <.0001
##  BC - TP      3.58 14.0 24.3  0.256  0.9940
##  BC - M     -80.81 11.4 21.9 -7.102  <.0001
##  TP - M     -84.39 14.4 25.2 -5.850  <.0001
##
## P value adjustment: tukey method for comparing a family of 4 estimates
## Tukey's post hoc comparisons: Slowwave amplitude
## ----------------------------------------------------
##  contrast estimate   SE   df t.ratio p.value
##  LN - BC     31.19 13.5 22.3  2.318  0.1241
##  LN - TP     35.56 17.1 26.6  2.085  0.1841
##  LN - M     -31.01 13.9 23.0 -2.230  0.1450
##  BC - TP      4.36 17.3 26.0  0.252  0.9942
##  BC - M     -62.20 14.2 22.4 -4.382  0.0012
##  TP - M     -66.56 17.7 27.2 -3.759  0.0043
##
## P value adjustment: tukey method for comparing a family of 4 estimates
```

3 Morphological parameters (N2 and N3 combined, no adjustment for electrode type)

3.1 Spike amplitude

3.1.1 Linear mixed model with Type II Wald chi-square tests

Table 15: LMM (Type II Wald chi-square tests)

	Chisq	Df	Pr(>Chisq)
Area	90.864949	2	0.0000000
Sleep	4.416303	3	0.2198785
SOZ	5.466402	1	0.0193855
Area:Sleep	3.582879	6	0.7329124
Area:SOZ	3.208001	2	0.2010905
Sleep:SOZ	1.737182	3	0.6286992
Area:Sleep:SOZ	2.144348	6	0.9059556

3.1.2 Tukey's post hoc comparisons

Table 16: Marginal mean differences between brain areas by wake/sleep stage

contrast	Sleep	estimate	SE	df	t.ratio	p.value
LN - TP	N2-N3	39.61387	19.80598	140.1375	2.000096	0.115944
LN - M	N2-N3	-120.90410	14.22502	140.3472	-8.499400	0.000000
TP - M	N2-N3	-160.51797	20.84829	141.9106	-7.699336	0.000000
LN - TP	N1	39.61387	19.80598	140.1375	2.000096	0.115944
LN - M	N1	-120.90410	14.22502	140.3472	-8.499400	0.000000
TP - M	N1	-160.51797	20.84829	141.9106	-7.699336	0.000000
LN - TP	W	39.61387	19.80598	140.1375	2.000096	0.115944
LN - M	W	-120.90410	14.22502	140.3472	-8.499400	0.000000
TP - M	W	-160.51797	20.84829	141.9106	-7.699336	0.000000
LN - TP	REM	39.61387	19.80598	140.1375	2.000096	0.115944
LN - M	REM	-120.90410	14.22502	140.3472	-8.499400	0.000000
TP - M	REM	-160.51797	20.84829	141.9106	-7.699336	0.000000

contrast	estimate	SE	df	t.ratio	p.value
LN - TP	39.61387	19.80598	140.1375	2.000096	0.115944
LN - M	-120.90410	14.22502	140.3472	-8.499400	0.000000
TP - M	-160.51797	20.84829	141.9106	-7.699336	0.000000

contrast	estimate	SE	df	t.ratio	p.value
nSOZ - SOZ	-30.25903	12.80587	136.8598	-2.362903	0.0195399

3.2 Spike sharpness

3.2.1 Linear mixed model with Type II Wald chi-square tests

Table 19: LMM (Type II Wald chi-square tests)

	Chisq	Df	Pr(>Chisq)
Area	216.3206017	2	0.0000000
Sleep	5.4334860	3	0.1426715
SOZ	0.1313164	1	0.7170711
Area:Sleep	2.6013739	6	0.8569543
Area:SOZ	21.3267739	2	0.0000234
Sleep:SOZ	2.1757580	3	0.5367391
Area:Sleep:SOZ	0.7648871	6	0.9929823

3.2.2 Tukey's post hoc comparisons

Table 20: Marginal mean differences between brain areas by wake/sleep stage

contrast	Sleep	estimate	SE	df	t.ratio	p.value
LN - TP	N2-N3	-12.656883	14.56240	119.6990	-0.8691483	0.6607488
LN - M	N2-N3	-84.860349	10.40736	120.3169	-8.1538821	0.0000000
TP - M	N2-N3	-72.203466	14.98704	120.7123	-4.8177279	0.0000127
LN - TP	N1	-11.157525	14.56240	119.6990	-0.7661874	0.7244221
LN - M	N1	-83.042969	10.40736	120.3169	-7.9792575	0.0000000
TP - M	N1	-71.885444	14.98704	120.7123	-4.7965081	0.0000138
LN - TP	W	-9.355234	14.56240	119.6990	-0.6424241	0.7969944
LN - M	W	-65.714353	10.40736	120.3169	-6.3142220	0.0000000
TP - M	W	-56.359119	14.98704	120.7123	-3.7605245	0.0007639
LN - TP	REM	-13.784241	15.64678	119.1461	-0.8809635	0.6533392
LN - M	REM	-75.781484	11.09518	118.9127	-6.8301259	0.0000000
TP - M	REM	-61.997243	15.62050	120.8200	-3.9689673	0.0003598

Table 21: Marginal mean differences between brain areas by SOZ

contrast	SOZ	estimate	SE	df	t.ratio	p.value
LN - TP	nSOZ	-41.57546	12.111481	124.1409	-3.432731	0.0023281
LN - M	nSOZ	-71.52042	6.894271	119.7687	-10.373891	0.0000000
TP - M	nSOZ	-29.94496	12.589167	124.8030	-2.378629	0.0491963
LN - TP	SOZ	18.09852	9.724244	120.8186	1.861175	0.1545173
LN - M	SOZ	-83.17916	8.548677	124.9191	-9.730062	0.0000000
TP - M	SOZ	-101.27768	10.305904	125.5233	-9.827151	0.0000000

Table 22: Marginal mean differences between SOZ and non SOZ by brain area

contrast	Area	estimate	SE	df	t.ratio	p.value
nSOZ - SOZ	LN	-4.207713	7.478677	120.9684	-0.5626279	0.5747295
nSOZ - SOZ	TP	55.466262	13.611336	123.1070	4.0750050	0.0000819
nSOZ - SOZ	M	-15.866456	7.634207	119.9382	-2.0783373	0.0398110

contrast	estimate	SE	df	t.ratio	p.value
LN - TP	-11.73847	7.905723	124.2430	-1.484807	0.301686
LN - M	-77.34979	5.602585	124.7780	-13.806089	0.000000
TP - M	-65.61132	8.339437	126.2031	-7.867596	0.000000

3.3 Slow wave amplitude

3.3.1 Linear mixed model with Type II chhi-square tests

Table 24: LMM (Type II Wald chi-square tests)

	Chisq	Df	Pr(>Chisq)
Area	29.6477334	2	0.0000004
Sleep	1.8578864	3	0.6024199
SOZ	2.0567230	1	0.1515357
Area:Sleep	1.7400777	6	0.9419855
Area:SOZ	0.7202750	2	0.6975804
Sleep:SOZ	0.4677943	3	0.9259077
Area:Sleep:SOZ	2.1459069	6	0.9058022

3.3.2 Tukey's post hoc comparisons

contrast	estimate	SE	df	t.ratio	p.value
LN - TP	25.12031	11.405185	126.9827	2.202534	0.0745941
LN - M	-29.65556	8.056379	126.9970	-3.681003	0.0009917
TP - M	-54.77586	11.946707	123.4902	-4.585018	0.0000325

contrast	Sleep	estimate	SE	df	t.ratio	p.value
LN - TP	N2-N3	32.76793	21.20902	122.0829	1.5449998	0.2736845
LN - M	N2-N3	-36.62033	15.13075	122.4563	-2.4202583	0.0444581
TP - M	N2-N3	-69.38826	21.78775	123.8516	-3.1847370	0.0051763
LN - TP	N1	15.75088	21.20902	122.0829	0.7426501	0.7386250
LN - M	N1	-32.22788	15.13075	122.4563	-2.1299590	0.0880130
TP - M	N1	-47.97876	21.78775	123.8516	-2.2020978	0.0747663
LN - TP	W	27.59720	21.20902	122.0829	1.3012010	0.3972537
LN - M	W	-21.10813	15.13075	122.4563	-1.3950486	0.3466441
TP - M	W	-48.70533	21.78775	123.8516	-2.2354455	0.0692403
LN - TP	REM	24.36521	22.81521	121.3521	1.0679375	0.5357635
LN - M	REM	-28.66589	16.18350	120.8406	-1.7713037	0.1836136
TP - M	REM	-53.03110	22.70628	124.0840	-2.3355260	0.0546479

contrast	SOZ	estimate	SE	df	t.ratio	p.value
LN - TP	nSOZ	20.82518	17.48829	126.9551	1.190807	0.4608232
LN - M	nSOZ	-35.79407	10.03788	121.9549	-3.565899	0.0014962
TP - M	nSOZ	-56.61925	18.14793	126.4481	-3.119873	0.0063032
LN - TP	SOZ	29.41543	14.13372	123.8768	2.081224	0.0979091
LN - M	SOZ	-23.51705	12.29029	126.9394	-1.913465	0.1390452
TP - M	SOZ	-52.93248	14.80455	125.7572	-3.575419	0.0014341

3.4 Spike duration

3.4.1 Linear mixed model with Type II Wald chi-square tests

Table 28: LMM (Type II chi-square tests)

	Chisq	Df	Pr(>Chisq)
Area	28.306214	2	0.0000007
Sleep	1.629211	3	0.6527847
SOZ	2.580573	1	0.1081825
Area:Sleep	11.966136	6	0.0627285
Area:SOZ	39.601677	2	0.0000000
Sleep:SOZ	1.444361	3	0.6951700
Area:Sleep:SOZ	7.712779	6	0.2599100

3.4.2 Tukey's post hoc comparisons

Table 29: Marginal mean differences between brain areas by SOZ

contrast	SOZ	estimate	SE	df	t.ratio	p.value
LN - TP	nSOZ	23.758417	4.251632	123.0224	5.588070	0.0000004
LN - M	nSOZ	6.694497	2.417061	119.3107	2.769685	0.0177556
TP - M	nSOZ	-17.063920	4.420444	123.6386	-3.860227	0.0005294
LN - TP	SOZ	-3.658359	3.410161	120.1718	-1.072782	0.5327717
LN - M	SOZ	14.395922	3.003363	123.9037	4.793267	0.0000137
TP - M	SOZ	18.054281	3.620744	124.4132	4.986346	0.0000060

Table 30: Marginal mean differences between SOZ and non SOZ by Area

contrast	Area	estimate	SE	df	t.ratio	p.value
nSOZ - SOZ	LN	-2.400023	2.622852	120.3060	-0.9150434	0.3619992
nSOZ - SOZ	TP	-29.816799	4.775800	122.0537	-6.2433098	0.0000000
nSOZ - SOZ	M	5.301401	2.676288	119.3961	1.9808783	0.0499038

Table 31: Marginal mean differences between brain areas by wake/sleep stage

contrast	Sleep	estimate	SE	df	t.ratio	p.value
LN - TP	N2-N3	8.679591	5.105019	119.2244	1.7002073	0.2093496
LN - M	N2-N3	10.987702	3.649674	119.8117	3.0105985	0.0088640
TP - M	N2-N3	2.308111	5.255314	120.0572	0.4391957	0.8992362
LN - TP	N1	14.630118	5.105019	119.2244	2.8658302	0.0135387
LN - M	N1	6.532049	3.649674	119.8117	1.7897624	0.1773717
TP - M	N1	-8.098069	5.255314	120.0572	-1.5409296	0.2755913
LN - TP	W	11.999850	5.105019	119.2244	2.3505985	0.0528394
LN - M	W	8.554562	3.649674	119.8117	2.3439253	0.0536796
TP - M	W	-3.445288	5.255314	120.0572	-0.6555817	0.7895660
LN - TP	REM	4.890558	5.483994	118.7403	0.8917876	0.6465391
LN - M	REM	16.106526	3.888530	118.5554	4.1420597	0.0001904
TP - M	REM	11.215968	5.477533	120.1414	2.0476314	0.1054047

contrast	estimate	SE	df	t.ratio	p.value
LN - TP	10.0500292	2.775706	123.1593	3.6207106	0.0012359
LN - M	10.5452098	1.968348	123.7943	5.3573909	0.0000012
TP - M	0.4951806	2.931101	125.1574	0.1689401	0.9843907

3.5 Slow wave duration

3.5.1 Linear mixed model with Type II Wald chi-square tests

Table 33: LMM (Type II Wald chi-square tests)

	Chisq	Df	Pr(>Chisq)
Area	1.8962337	2	0.3874700
Sleep	3.7032844	3	0.2953380
SOZ	0.4892882	1	0.4842450
Area:Sleep	15.1248495	6	0.0193076
Area:SOZ	8.5626780	2	0.0138241
Sleep:SOZ	0.2480581	3	0.9694817
Area:Sleep:SOZ	5.5284450	6	0.4780254

3.5.2 Tukey's post hoc comparisons

Table 34: Marginal mean differences between areas by SOZ

contrast	SOZ	estimate	SE	df	t.ratio	p.value
LN - TP	nSOZ	4.911662	10.315938	123.5221	0.4761236	0.8826769
LN - M	nSOZ	-3.797015	5.867855	119.5114	-0.6470874	0.7943717
TP - M	nSOZ	-8.708677	10.724371	124.1648	-0.8120454	0.6962885
LN - TP	SOZ	-4.995926	8.277810	120.4548	-0.6035324	0.8184583
LN - M	SOZ	18.661478	7.284671	124.3680	2.5617462	0.0309638
TP - M	SOZ	23.657405	8.782137	124.9265	2.6938097	0.0217589

Table 35: Marginal mean differences between SOZ and non SOZ by brain area

contrast	Area	estimate	SE	df	t.ratio	p.value
nSOZ - SOZ	LN	-11.20895	6.366514	120.5965	-1.760610	0.0808395
nSOZ - SOZ	TP	-21.11653	11.590177	122.5170	-1.821934	0.0709043
nSOZ - SOZ	M	11.24955	6.497380	119.6311	1.731398	0.0859588

Table 36: Marginal mean differences between brain areas by wake/sleep stage

contrast	Sleep	estimate	SE	df	t.ratio	p.value
LN - TP	N2-N3	25.447517	12.393797	119.4310	2.0532462	0.1041696
LN - M	N2-N3	6.167300	8.859242	120.0353	0.6961431	0.7661975
TP - M	N2-N3	-19.280217	12.757199	120.3429	-1.5113205	0.2892305
LN - TP	N1	6.527552	12.393797	119.4310	0.5266790	0.8584321
LN - M	N1	7.564037	8.859242	120.0353	0.8538018	0.6703527
TP - M	N1	1.036484	12.757199	120.3429	0.0812470	0.9963674
LN - TP	W	-31.086246	12.393797	119.4310	-2.5082100	0.0356908
LN - M	W	2.653362	8.859242	120.0353	0.2995022	0.9517764
TP - M	W	33.739608	12.757199	120.3429	2.6447504	0.0249453
LN - TP	REM	-1.057354	13.315081	118.9156	-0.0794102	0.9965295
LN - M	REM	13.344228	9.441507	118.7099	1.4133578	0.3372976
TP - M	REM	14.401581	13.296537	120.4372	1.0831077	0.5263675

Table 37: Marginal mean differences between wake/sleep stages by brain area

contrast	Area	estimate	SE	df	t.ratio	p.value
N2-N3 - N1	LN	8.778594	8.328374	117.0348	1.0540585	0.7179818
N2-N3 - W	LN	17.214823	8.328374	117.0348	2.0670089	0.1700727
N2-N3 - REM	LN	1.008661	9.106134	118.5551	0.1107672	0.9995111
N1 - W	LN	8.436229	8.328374	117.0348	1.0129503	0.7421916
N1 - REM	LN	-7.769933	9.106134	118.5551	-0.8532637	0.8287321
W - REM	LN	-16.206162	9.106134	118.5551	-1.7796974	0.2882054
N2-N3 - N1	TP	-10.141371	14.917635	117.0348	-0.6798243	0.9045734
N2-N3 - W	TP	-39.318940	14.917635	117.0348	-2.6357355	0.0463872
N2-N3 - REM	TP	-25.496210	15.354333	117.1864	-1.6605221	0.3492530
N1 - W	TP	-29.177569	14.917635	117.0348	-1.9559112	0.2108144
N1 - REM	TP	-15.354839	15.354333	117.1864	-1.0000330	0.7496605
W - REM	TP	13.822730	15.354333	117.1864	0.9002495	0.8047015
N2-N3 - N1	M	10.175331	8.914992	117.0348	1.1413729	0.6647581
N2-N3 - W	M	13.700886	8.914992	117.0348	1.5368365	0.4189675
N2-N3 - REM	M	8.185588	8.914992	117.0348	0.9181824	0.7951900
N1 - W	M	3.525555	8.914992	117.0348	0.3954636	0.9789402
N1 - REM	M	-1.989742	8.914992	117.0348	-0.2231906	0.9960573
W - REM	M	-5.515297	8.914992	117.0348	-0.6186542	0.9259393

4 Morphological parameters (N2 and N3 combined)

4.1 Spike amplitude

4.1.1 Linear mixed model with Type II Wald chi-square tests

Table 38: LMM (Type II Wald chi-square tests)

	Chisq	Df	Pr(>Chisq)
Area	28.548260	2	0.0000006
Sleep	4.333708	3	0.2276115
SOZ	7.936348	1	0.0048452
Type	10.916828	2	0.0042603
Area:Sleep	4.054492	6	0.6693022
Area:SOZ	4.056949	2	0.1315360
Sleep:SOZ	2.361433	3	0.5008541
Area:Sleep:SOZ	3.147503	6	0.7901190

4.1.2 Tukey's post hoc comparisons

contrast	estimate	SE	df	t.ratio	p.value
LN - TP	33.31805	19.85027	193.5177	1.678469	0.2160657
LN - M	-71.71694	16.55287	195.1488	-4.332600	0.0000698
TP - M	-105.03499	21.69250	194.6725	-4.841996	0.0000078

contrast	estimate	SE	df	t.ratio	p.value
nSOZ - SOZ	-34.09979	12.07144	190.3157	-2.824833	0.0052348

contrast	estimate	SE	df	t.ratio	p.value
depth - grid	32.44956	25.19217	196.5760	1.288081	0.4035742
depth - strip	54.80862	17.57281	196.5108	3.118944	0.0058938
grid - strip	22.35906	18.43299	194.2658	1.212992	0.4469231

4.2 Spike duration

4.2.1 Linear mixed model with Type II Wald chi-square tests

Table 42: LMM (Type II Wald chi-square tests)

	Chisq	Df	Pr(>Chisq)
Area	5.155959	2	0.0759273
Sleep	3.933158	3	0.2687729
SOZ	1.299451	1	0.2543135
Type	10.552576	2	0.0051114
Area:Sleep	9.453433	6	0.1496370
Area:SOZ	28.820861	2	0.0000006
Sleep:SOZ	0.874722	3	0.8315233
Area:Sleep:SOZ	6.426584	6	0.3771366

4.2.2 Tukey's post hoc comparisons

contrast	Area	estimate	SE	df	t.ratio	p.value
nSOZ - SOZ	LN	-7.687685	2.968769	180.8590	-2.589519	0.0103936
nSOZ - SOZ	TP	-23.993431	5.892540	182.8327	-4.071831	0.0000694
nSOZ - SOZ	M	8.337336	2.946531	182.2751	2.829543	0.0051841

contrast	SOZ	estimate	SE	df	t.ratio	p.value
LN - TP	nSOZ	15.132456	5.464084	185.1235	2.7694408	0.0169492
LN - M	nSOZ	1.917107	2.942386	183.4829	0.6515484	0.7917263
TP - M	nSOZ	-13.215349	5.520156	184.5750	-2.3940172	0.0462643
LN - TP	SOZ	-1.173291	3.903161	181.0068	-0.3006002	0.9514244
LN - M	SOZ	17.942128	4.015737	185.9757	4.4679545	0.0000406
TP - M	SOZ	19.115419	4.426452	185.9177	4.3184511	0.0000755

contrast	estimate	SE	df	t.ratio	p.value
depth - grid	-6.248772	4.119272	176.7297	-1.516960	0.2854205
depth - strip	3.007653	2.866868	178.2805	1.049107	0.5469692
grid - strip	9.256425	3.024964	185.7808	3.060012	0.0071392

4.3 Spike sharpness

4.3.1 Linear mixed model with Type II Wald chi-square tests

Table 46: LMM (Type II Wald chi-square tests)

	Chisq	Df	Pr(>Chisq)
Area	58.5264657	2	0.0000000
Sleep	6.6500250	3	0.0839300
SOZ	2.0373551	1	0.1534763
Type	9.1607162	2	0.0102512
Area:Sleep	1.1907273	6	0.9773402
Area:SOZ	22.8989705	2	0.0000107
Sleep:SOZ	2.5487120	3	0.4665520
Area:Sleep:SOZ	0.6828747	6	0.9948532

4.3.2 Tukey's post hoc comparisons

contrast	Area	estimate	SE	df	t.ratio	p.value
nSOZ - SOZ	LN	-3.924793	7.479893	180.3171	-0.5247124	0.6004277
nSOZ - SOZ	TP	56.559101	14.850762	182.1310	3.8084982	0.0001910
nSOZ - SOZ	M	-24.656030	7.426229	181.6713	-3.3201278	0.0010874

contrast	SOZ	estimate	SE	df	t.ratio	p.value
LN - TP	nSOZ	-41.916086	13.780632	184.5040	-3.0416664	0.0075626
LN - M	nSOZ	-43.780123	7.418010	182.8477	-5.9018692	0.0000001
TP - M	nSOZ	-1.864037	13.918238	183.8718	-0.1339276	0.9901607
LN - TP	SOZ	18.567807	9.834252	180.4454	1.8880751	0.1450614
LN - M	SOZ	-64.511361	10.134403	185.7157	-6.3655806	0.0000000
TP - M	SOZ	-83.079168	11.169643	185.5778	-7.4379427	0.0000000

contrast	estimate	SE	df	t.ratio	p.value
depth - grid	17.434128	10.425599	180.4782	1.6722422	0.2186856
depth - strip	21.642916	7.254263	181.5461	2.9834755	0.0090533
grid - strip	4.208789	7.632295	185.3529	0.5514447	0.8458656

4.4 Slow wave amplitude

4.4.1 Linear mixed model with Type II Wald chi-square tests

Table 50: LMM (Type II Wald chi-square tests)

	Chisq	Df	Pr(>Chisq)
Area	10.370555	2	0.0055984
Sleep	2.207771	3	0.5304194
SOZ	4.795098	1	0.0285408
Type	2.491359	2	0.2877453
Area:Sleep	1.707029	6	0.9445773
Area:SOZ	1.899807	2	0.3867784
Sleep:SOZ	1.399768	3	0.7055891
Area:Sleep:SOZ	1.176473	6	0.9780308

4.4.2 Tukey's post hoc comparisons

contrast	estimate	SE	df	t.ratio	p.value
LN - TP	18.90484	10.983650	196.8901	1.721180	0.1998339
LN - M	-19.36491	9.126474	196.6181	-2.121839	0.0880398
TP - M	-38.26974	11.975879	196.8635	-3.195569	0.0046131

contrast	estimate	SE	df	t.ratio	p.value
nSOZ - SOZ	-15.00783	6.709836	193.3143	-2.236691	0.0264491

4.5 Slow wave duration

4.5.1 Linear mixed model with Type II Wald chi-square tests

Table 53: LMM (Type II Wald chi-square tests)

	Chisq	Df	Pr(>Chisq)
Area	12.4831717	2	0.0019468
Sleep	6.0222639	3	0.1105320
SOZ	0.3841584	1	0.5353861
Type	10.0193712	2	0.0066730
Area:Sleep	12.4199344	6	0.0532301
Area:SOZ	5.2543048	2	0.0722840
Sleep:SOZ	0.8050492	3	0.8482589
Area:Sleep:SOZ	4.5699533	6	0.6000266

4.5.2 Tukey's post hoc comparisons

contrast	Area	estimate	SE	df	t.ratio	p.value
N2-N3 - N1	LN	18.6435076	10.36278	176.7041	1.7990830	0.2772204
N2-N3 - W	LN	26.3944796	10.26761	176.5355	2.5706550	0.0530393
N2-N3 - REM	LN	2.2913003	11.41414	182.2536	0.2007422	0.9971270
N1 - W	LN	7.7509720	10.36278	176.7041	0.7479624	0.8774342
N1 - REM	LN	-16.3522073	11.49997	182.2188	-1.4219349	0.4873141
W - REM	LN	-24.1031793	11.41414	182.2536	-2.1116944	0.1532434
N2-N3 - N1	TP	-7.3606209	18.97564	176.5427	-0.3878984	0.9801144
N2-N3 - W	TP	-39.2415378	18.97242	176.5355	-2.0683464	0.1675814
N2-N3 - REM	TP	-47.4442596	19.73972	177.0797	-2.4034917	0.0800995
N1 - W	TP	-31.8809169	18.97564	176.5427	-1.6800971	0.3372601
N1 - REM	TP	-40.0836386	19.74178	177.0911	-2.0303966	0.1807789
W - REM	TP	-8.2027217	19.73972	177.0797	-0.4155439	0.9757560
N2-N3 - N1	M	5.0755292	10.55094	176.7290	0.4810498	0.9632066
N2-N3 - W	M	10.6101904	10.44640	176.5355	1.0156789	0.7404875
N2-N3 - REM	M	4.3956100	10.45656	176.5430	0.4203687	0.9749394
N1 - W	M	5.5346612	10.55094	176.7290	0.5245656	0.9530635
N1 - REM	M	-0.6799192	10.56014	176.7597	-0.0643855	0.9999039
W - REM	M	-6.2145804	10.45656	176.5430	-0.5943237	0.9336858

contrast	Sleep	estimate	SE	df	t.ratio	p.value
LN - TP	N2-N3	27.671893	16.11017	185.4524	1.7176662	0.2012930
LN - M	N2-N3	29.658373	11.43553	184.9996	2.5935286	0.0275726
TP - M	N2-N3	1.986480	16.51929	185.9233	0.1202521	0.9920597
LN - TP	N1	1.667765	16.16502	185.4467	0.1031712	0.9941490
LN - M	N1	16.090395	11.58753	185.3494	1.3885953	0.3489439
TP - M	N1	14.422630	16.53265	185.8607	0.8723728	0.6583844
LN - TP	W	-37.964124	16.11017	185.4524	-2.3565317	0.0507652
LN - M	W	13.874084	11.43553	184.9996	1.2132437	0.4468492
TP - M	W	51.838208	16.51929	185.9233	3.1380402	0.0055894
LN - TP	REM	-22.063667	17.48380	184.3845	-1.2619491	0.4185439
LN - M	REM	31.762683	12.39260	185.6269	2.5630355	0.0299065
TP - M	REM	53.826350	17.37103	185.2738	3.0986282	0.0063316

contrast	Area	estimate	SE	df	t.ratio	p.value
nSOZ - SOZ	LN	-13.007967	7.977747	185.9493	-1.630531	0.1046819
nSOZ - SOZ	TP	-18.866883	15.773570	181.5632	-1.196107	0.2332144
nSOZ - SOZ	M	9.995572	7.877221	185.8742	1.268921	0.2060559

contrast	SOZ	estimate	SE	df	t.ratio	p.value
LN - TP	nSOZ	-4.742575	14.405109	173.5270	-0.3292287	0.9420236
LN - M	nSOZ	11.344615	7.820817	185.0835	1.4505664	0.3172982
TP - M	nSOZ	16.087190	14.618226	174.4129	1.1004885	0.5150552
LN - TP	SOZ	-10.601491	10.483692	185.9945	-1.0112364	0.5707036
LN - M	SOZ	34.348153	10.466028	159.9711	3.2818711	0.0036066
TP - M	SOZ	44.949644	11.557962	162.2838	3.8890632	0.0004286

contrast	estimate	SE	df	t.ratio	p.value
depth - grid	11.911050	10.208857	99.69068	1.166737	0.4757724
depth - strip	20.803257	7.127113	108.19336	2.918890	0.0118077
grid - strip	8.892208	7.921963	167.02807	1.122475	0.5015948

5 R session information

```
## R version 3.5.2 (2018-12-20)
## Platform: x86_64-pc-linux-gnu (64-bit)
## Running under: Linux Mint 19.1
##
## Matrix products: default
## BLAS: /opt/microsoft/ropen/3.5.2/lib64/R/lib/libRblas.so
## LAPACK: /opt/microsoft/ropen/3.5.2/lib64/R/lib/libRlapack.so
##
## locale:
##  [1] LC_CTYPE=en_GB.UTF-8       LC_NUMERIC=C
##  [3] LC_TIME=en_GB.UTF-8        LC_COLLATE=en_GB.UTF-8
##  [5] LC_MONETARY=fr_FR.UTF-8    LC_MESSAGES=en_GB.UTF-8
##  [7] LC_PAPER=fr_FR.UTF-8       LC_NAME=C
##  [9] LC_ADDRESS=C               LC_TELEPHONE=C
## [11] LC_MEASUREMENT=fr_FR.UTF-8 LC_IDENTIFICATION=C
##
## attached base packages:
## [1] grid      stats     graphics  grDevices utils     datasets  methods
## [8] base
##
## other attached packages:
##  [1] reshape2_1.4.3      readxl_1.2.0        emmeans_1.3.2
##  [4] multcompView_0.1-7  multcomp_1.4-8      TH.data_1.0-10
##  [7] MASS_7.3-51.5       survival_2.43-3     mvtnorm_1.0-8
## [10] lmerTest_3.0-1      lme4_1.1-19         Matrix_1.2-15
## [13] car_3.0-2           carData_3.0-2       Rmisc_1.5
## [16] lattice_0.20-38     plyr_1.8.4          data.table_1.12.2
## [19] knitr_1.22          ggsci_2.9           RColorBrewer_1.1-2
## [22] gridExtra_2.3       ggplot2_3.2.1       RevoUtils_11.0.2
## [25] RevoUtilsMath_11.0.0
##
## loaded via a namespace (and not attached):
##  [1] splines_3.5.2     highr_0.8         cellranger_1.1.0
##  [4] yaml_2.2.0        numDeriv_2016.8-1 pillar_1.5.1
##  [7] glue_1.4.2        digest_0.6.18     minqa_1.2.4
## [10] colorspace_1.4-1  sandwich_2.5-0    htmltools_0.3.6
## [13] pkgconfig_2.0.2   haven_2.0.0       purrr_0.3.2
## [16] xtable_1.8-4      scales_1.0.0      openxlsx_4.1.0
## [19] rio_0.5.16        tibble_3.1.0      generics_0.0.2
## [22] ellipsis_0.3.0    withr_2.1.2       lazyeval_0.2.2
## [25] pbkrtest_0.4-7    magrittr_1.5      crayon_1.3.4
## [28] estimability_1.3  evaluate_0.13     fansi_0.4.0
## [31] nlme_3.1-137      forcats_0.3.0     foreign_0.8-71
## [34] tools_3.5.2       hms_0.4.2         lifecycle_1.0.0
## [37] stringr_1.4.0     munsell_0.5.0     zip_1.0.0
## [40] compiler_3.5.2    rlang_0.4.10      nloptr_1.2.1
## [43] rmarkdown_1.11    gtable_0.3.0      codetools_0.2-15
## [46] abind_1.4-5       DBI_1.0.0         curl_3.3
## [49] R6_2.3.0          zoo_1.8-7         dplyr_1.0.5
## [52] utf8_1.1.4        stringi_1.2.4     parallel_3.5.2
## [55] Rcpp_1.0.3        vctrs_0.3.7       tidyselect_1.1.0
## [58] xfun_0.6          coda_0.19-2
```
