# Supplementary material for: Interictal epileptiform discharges show distinct spatiotemporal and morphological patterns across wake and sleep
Source: Brain Commun. 2022 Jul 18;4(5):fcac183. doi: 10.1093/braincomms/fcac183 (PMC9724782; doi:10.1093/braincomms/fcac183)
Supplement: fcac183_Supplementary_Data [file fcac183_Supplementary_Data.zip › Supplementary Tables and legends.pdf]

Supplementary Tables

Supplementary Table 1: Precision of the automatic IED detection method

**Patient Number**	**Stage of the sleep/wake cycle**					**Average per patient**
	**Awake**	**N1**	**N2**	**N3**	**REM**	
1	0.8	0.6	0.7	0.8	0.8	0.74
2	0.9	1.0	1.0	0.9	1.0	0.96
3	0.9	1.0	1.0	0.7	0.9	0.90
4	1.0	1.0	0.9	0.9	1.0	0.96
5	0.9	1.0	0.9	1.0	0.8	0.92
6	0.7	0.7	0.9	1.0	1.0	0.86
7	1.0	1.0	1.0	1.0	1.0	1.0
8	0.9	1.0	0.9	0.9	0.9	0.92
9	0.9	0.8	1.0	1.0	0.9	0.92
10	1.0	1.0	0.8	0.9		0.92
11	1.0	0.9	1.0	1.0	1.0	0.98
**Average per sleep/wake stage**	0.90	0.90	0.92	0.92	0.93	

Abbreviations: N1, N2 and N3: non-REM sleep stages; REM: rapid eye movement sleep.

Supplementary Table 2: Number of detected IEDs per brain area in each patient group

Patient Group	Brain area				Total
	Lateral neocortical	Mesial	Temporal pole	Basal cortical	
**M-SOZ**	90 892	113 384	60 543	29 604	294 423
**LN-SOZ**	79 152	8850	15 426	7024	110 452
**All patients**	170 044	122 234	75 969	36 628	404 875

Abbreviations: M-SOZ group, patients with a mesial seizure onset zone; LN-SOZ group, patients with a lateral neocortical seizure onset zone

Supplementary Table 3: The distribution of values extracted from EEG signals for each morphological parameter.

								outliers*	
	**Total N.**	**Minimum**	**Maximum**	**First quartile (Q1, 25%)**	**Median (Q2, 50%)**	**Third quartile (Q3, 75%)**	**Interquartile range (IQR)**	**N.**	**Percentage**
**Spike amplitude**	404,374	0	5,985.1	280.4	376.4	529.5	249.1	2,654	0.7
**Spike duration**	404,374	0	859	63	78	109	46	4,199	1
**Spike sharpness**	404,374	0	3,472.2	79.1	116	184.4	105.3	7,438	1.8
**Slow wave amplitude**	404,374	0	3,283.5	38.3	104	201.5	163.2	1,352	0.3
**Slow wave duration**	404,374	0	689	58	104	185	127	0	0

***outlier removal formula: all values > Q2 + 5*IQR**
