# Supplementary figures and images for: Interictal epileptiform discharges show distinct spatiotemporal and morphological patterns across wake and sleep
Source: Brain Commun. 2022 Jul 18;4(5):fcac183. doi: 10.1093/braincomms/fcac183 (PMC9724782; doi:10.1093/braincomms/fcac183)

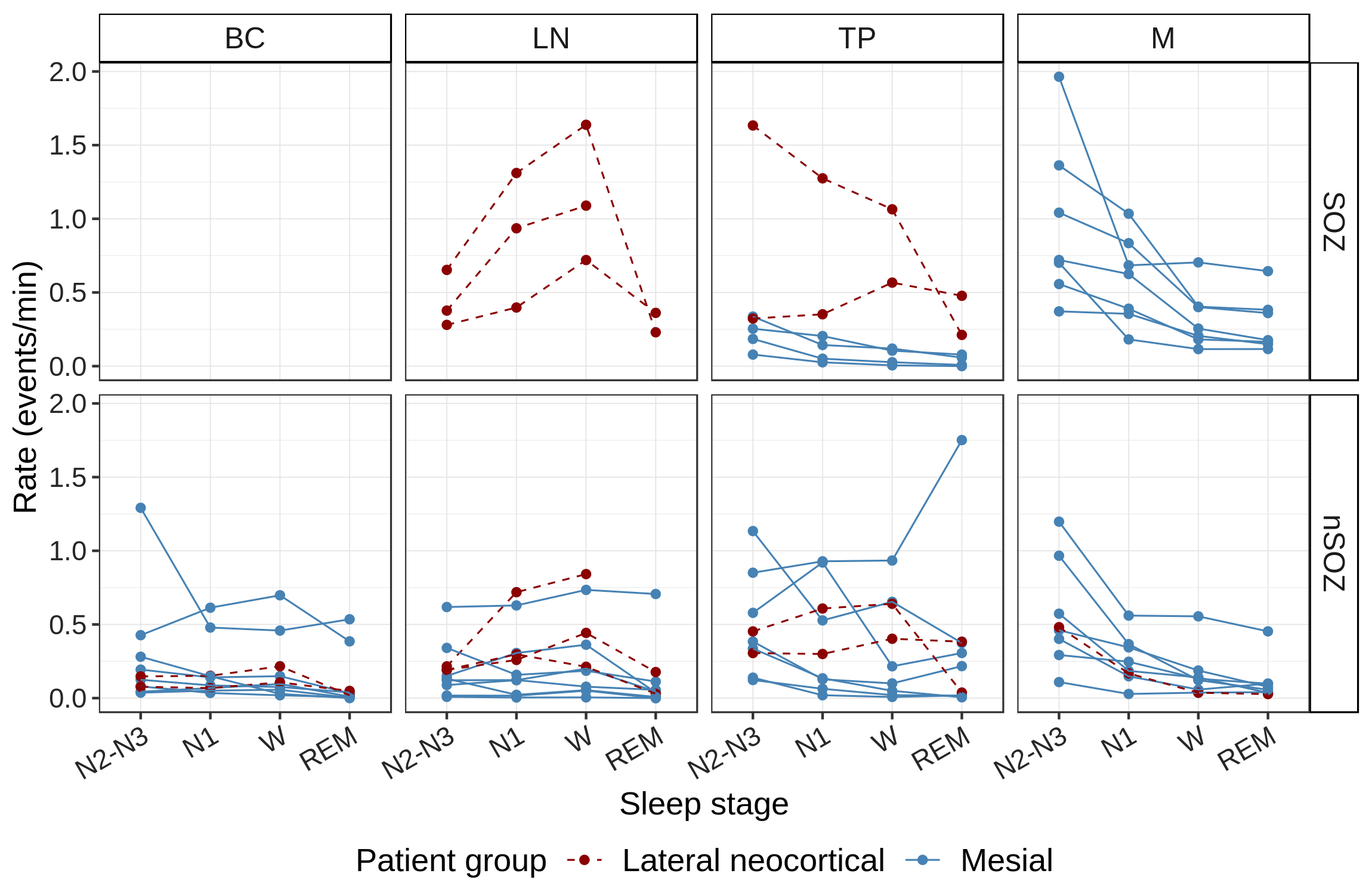

Supplement: fcac183_Supplementary_Data [file fcac183_Supplementary_Data.zip › Supplementary Fig. 3. The distribution of the rate of IEDs in patient groups during wake and sleep .tiff]

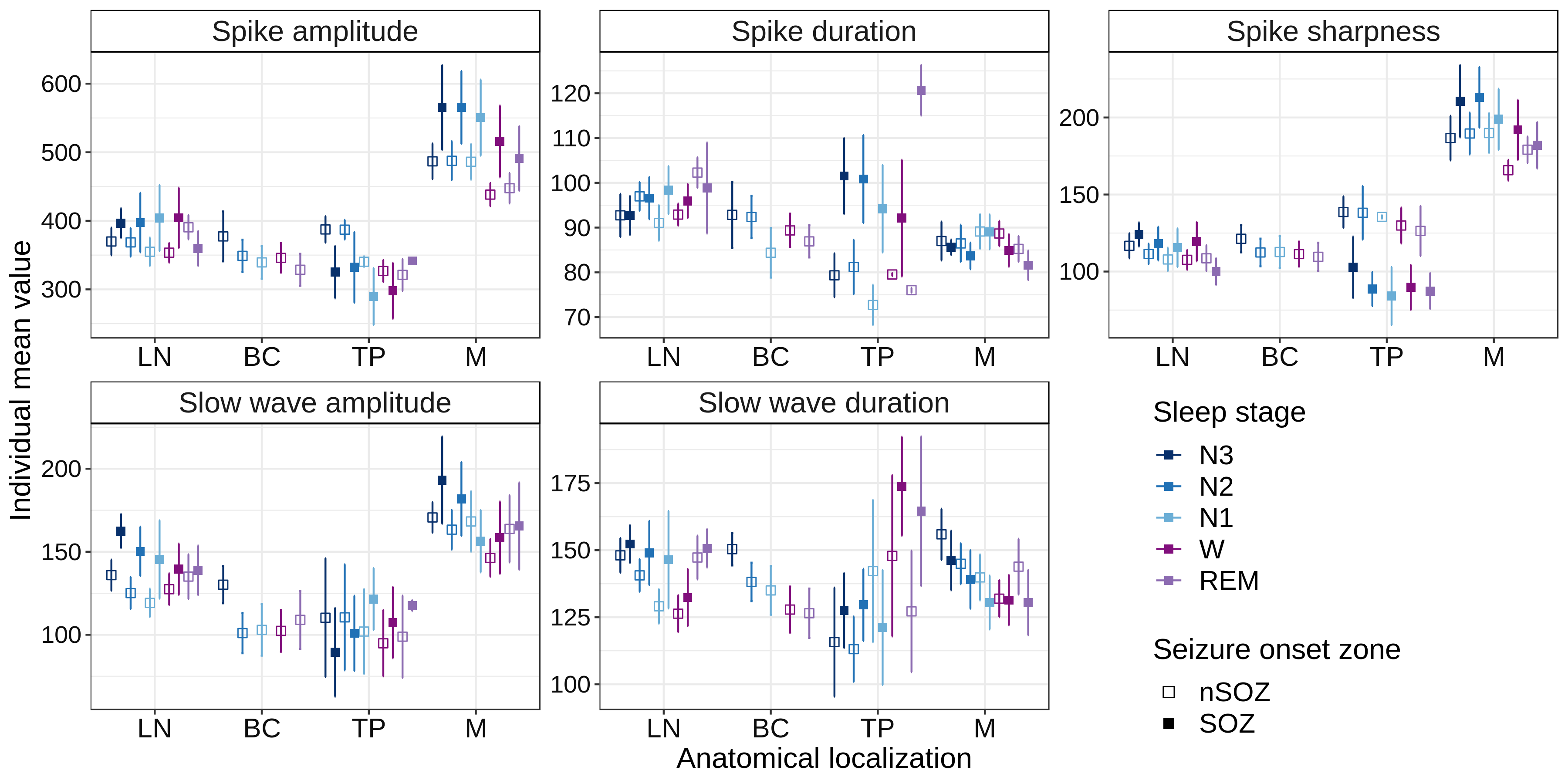

Supplement: fcac183_Supplementary_Data [file fcac183_Supplementary_Data.zip › Supplementary Fig. 4.morphological caracteristics during wake and sleep-N2 and N3 separated.tiff]
